# Design and synthesis of some new benzoylthioureido phenyl derivatives targeting carbonic anhydrase enzymes

**DOI:** 10.1080/14756366.2022.2126463

**Published:** 2022-09-27

**Authors:** Mazin A. A. Najm, Walaa R. Mahmoud, Azza T. Taher, Safinaz E-S. Abbas, Fadi M. Awadallah, Heba Abdelrasheed Allam, Daniela Vullo, Claudiu T. Supuran

**Affiliations:** a Department of Pharmaceutical Chemistry, College of Pharmacy, Al-Ayen University, Thi-Qar, Iraq; b Department of Pharmaceutical Chemistry, Faculty of Pharmacy, Cairo University, Cairo, Egypt; c Department of Pharmaceutical Organic Chemistry, Faculty of Pharmacy, Cairo University, Cairo, Egypt; d Department of Pharmaceutical Organic Chemistry, Faculty of Pharmacy, October 6 University (O6U), Giza, Egypt; e Department of NEUROFARBA, Section of Pharmaceutical and Nutraceutical Sciences, University of Florence, Firenze, Italy

**Keywords:** Sulphonamides, carbonic anhydrase, SLC-0111, benzoylthioureido derivatives

## Abstract

The present study aimed to develop potent carbonic anhydrase inhibitors (CAIs). The design of the target compounds was based on modifying the structure of the ureido-based carbonic anhydrase inhibitor SLC-0111. Six series of a substituted benzoylthioureido core were prepared featuring different zinc-binding groups; the conventional sulphamoyl group **4a–d** and **12a–c**, its bioisosteric carboxylic acid group **5a–d** and **13a–c** or the ethyl carboxylate group **6a–d** and **14a–c** as potential prodrugs. All compounds were assessed for their carbonic anhydrase (CA) inhibitory activity against a panel of four physiologically relevant human CA isoforms hCA I and hCA II, and hCA IX, and hCA XII. Compounds **4a**, **4b**, **4c, 4d, 5d**, **12a**, and **12c** revealed significant inhibitory activity against hCA I that would highlight these compounds as promising drug candidates for the treatment of glaucoma.

## Introduction

Carbonic anhydrase (CA, EC 4.2.1.1) enzyme is a well-known protein that exists in humans as fifteen distinct isoforms of the α-genetic family^1^. CAs enzymes are metalloproteins containing zinc that efficiently catalyses the reversible conversion of carbon dioxide to bicarbonate and release proton^2^. CA enzyme has eight distinct classes (α, β, γ, δ, ζ, η, θ, and ι) that have no significant sequence identity and were discovered independently. As a result, the carbonic anhydrase classes are excellent examples of catalytic function of biological evolution^3^. CAs are involved in important physiological processes such as acid–base regulation, gluconeogenesis, biosynthetic reactions^4^, electrolyte secretion, bone resorption/calcification, and tumorigenicity^5^. Consequently, inhibition of CAs can be a target for the treatment of glaucoma, obesity, neuropathic pain, arthritis, Alzheimer’s disease and cancer^6^^,^^7^. Some CA isoforms notably isoform IX are found in high concentrations in many solid cancers and in lower concentrations in a variety of normal tissues with strong correlation between its expression and hypoxia^8–10^ and where high CA levels are associated with poor prognosis^11^. Therefore, the design of CAIs have been a beneficial strategy for the treatment of many diseases since the late 1950s where the primary sulphonamides and their isosteres emerged as promising drugs for decades^12–14^. Most of the potent CAIs have been correlated with the presence of suitable zinc binding group (ZBG) to establish the required interaction within the hCAs active sites^15^, nevertheless many non-zinc binding CAIs have been recently developed^16^.

**Figure 1.**
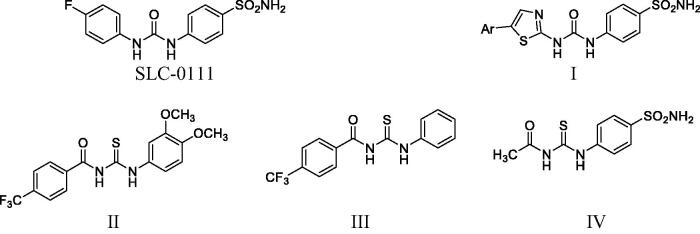
Chemical structure of some ureido and thioureido CAIs.

Literature survey unveiled that many ureido and thioureido phenyl derivatives exhibit remarkable CA inhibition activity (Figure 1). Interestingly, the ureido-substituted benzenesulfonamide CA inhibitor SLC-0111 was developed with a highly effective hCA IX/XII inhibitory activity and it was progressed to phase I/II clinical trials for the treatment of advanced metastatic solid cancers^17^^,^^18^. Many studies focussed on the development of various SLC-0111 analogues through replacement of the 4-fluorophenyl tail, with either substituted thiazole compound **I**^19^, 4-trifluoromethylbenzoyl compounds **II** and **III** (IC_50_ = 1.90 and 2.48 µM) respectively against hCAII^20^ or acetyl moiety compound **IV (**IC_50_ = 2.14 µM) against hCAII^21^ where they showed significant carbonic anhydrase inhibitory activity.

Accordingly, built on the reported carbonic anhydrase inhibitory activity of SLC-0111 and substituted benzoylthioureido compounds II–IV, we planned to synthesise new benzoylthioureido derivatives with potential carbonic anhydrase inhibitory activity. This is attained via replacement of 4-fluorophenyl ureido moiety of SLC-0111 with un/substituted benzoylthioureido ones, where the substitution at the benzoyl moiety involves either 3-chloro, 3,4-dichloro or 3-bromo substituent while retaining sulphamoyl phenyl of SLC-0111 to produce compounds **4a–d**, or bioisosteric replacement of the sulphamoyl phenyl with benzoic acid to obtain **5a–d**, or with ethyl benzoate moiety as potential prodrugs to give **6a–d**.

Further modification includes substitution of 4-fluorophenyl ureido of SLC-0111 with 2-methoxy-4-substituted benzamido moiety while keeping the same substitution pattern of the zinc binding groups (sulphamoyl phenyl or benzoic acid) to afford **12a–c** and **13a–c**, respectively, or via the prodrug moiety (ethyl benzoate) to obtain **14a–c** (Table 1).

**Table 1.** Inhibition data (K_I_, nM) of human CA isoforms hCA I, II, IX and XII with compounds **4a–d, 5a–d**, **6a–d, 12a–c, 13a–c,** and **14a–c** against **SLC-0111** and **AAZ**; by a stopped flow CO_2_ hydrase assay.

				K_I_ (nM)^a^			
Compound	*R*	*R*_1_	*R*_2_	hCA I	hCA II	hCA IX	hCA XII
**4a**	**–**	**H**	**H**	75.70	78.00	106.00	57.40
**4b**	**–**	**Cl**	**H**	58.50	58.10	115.40	>100,000
**4c**	**–**	**Cl**	**Cl**	40.40	90.00	>100,000	88.10
**4d**	**–**	**Br**	**H**	65.00	67.00	>100000	82.00
**5a**	**–**	**H**	**H**	1504.70	464.60	2932.00	>100,000
**5b**	**–**	**Cl**	**H**	8797.60	5535.80	>100,000	>100,000
**5c**	**–**	**Cl**	**Cl**	>100,000	6000.30	1741.00	>100,000
**5d**	**–**	**Br**	**H**	5556.20	>100,000	>100,000	>100,000
**6a**	**–**	**H**	**H**	>100,000	>100,000	1739.30	>100,000
**6b**	**–**	**Cl**	**H**	>100,000	>100,000	1403.60	>100,000
**6c**	**–**	**Cl**	**Cl**	809.10	>100,000	239.10	>100,000
**6d**	**–**	**Br**	**H**	85.38	31.00	>100,000	>100,000
**12a**	**-CH_3_**	**–**	**–**	67.60	15.50	32.20	83.00
**12b**	**-CH(CH_3_)_2_**	**–**	**–**	>100,000	2766.20	1784.30	>100,000
**12c**	**-*p*-CH_3_-C_6_H_4_**	**–**	**–**	91.00	94.20	144.00	225.70
**13a**	**-CH_3_**	**–**	**–**	1709.60	647.00	1092.60	>100,000
**13b**	**-CH(CH_3_)_2_**	**–**	**–**	>100,000	193.50	289.00	>100,000
**13c**	**-*p*-CH_3_-C_6_H_4_**	**–**	**–**	>100,000	504.00	802.10	>100,000
**14a**	**-CH_3_**	**–**	**–**	>100,000	>100,000	1146.20	>100,000
**14b**	**-CH(CH_3_)_2_**	**–**	**–**	>100,000	>100,000	1496.40	>100,000
**14c**	**-*p*-CH_3_-C_6_H_4_**	**–**	**–**	>100,000	2019.70	1367.30	>100,000
**SLC-0111^23^**	**–**	**–**	**–**	5080.00	960.00	45.00	4.50
**AAZ**	**–**	**–**	**–**	250.00	12.1	25.70	5.70

^a^Mean from 3 different assays, by a stopped flow technique (errors were in the range of ± 5–10% of the reported values).

**Figure 2.**
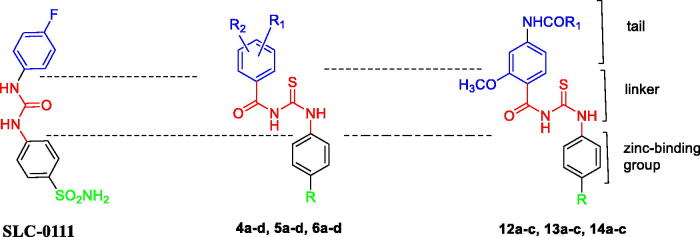
Design of the target compounds as analogues to SCL-0111.

This amendment aimed to explore the effect of such modification on the potency and/or selectivity of the designed compounds. Meanwhile, a highly flexible carbonyl thioureido linker was retained in all compounds (Figure 2).

## Results and discussion

### Chemistry

**Scheme 1.**
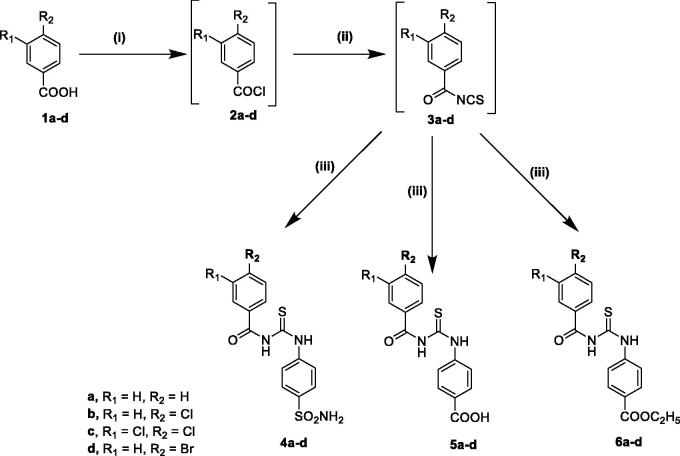
Reagents and reaction conditions: (i) SOCl_2,_ methylene chloride, reflux, 4–5 h, (ii) NH_4_SCN, acetone, reflux, 1–3 h, (iii) sulphanilamide or 4-aminobenzoic acid or ethyl 4-aminobenzoate, acetone, reflux, 2–3 h.

**Scheme 2.**
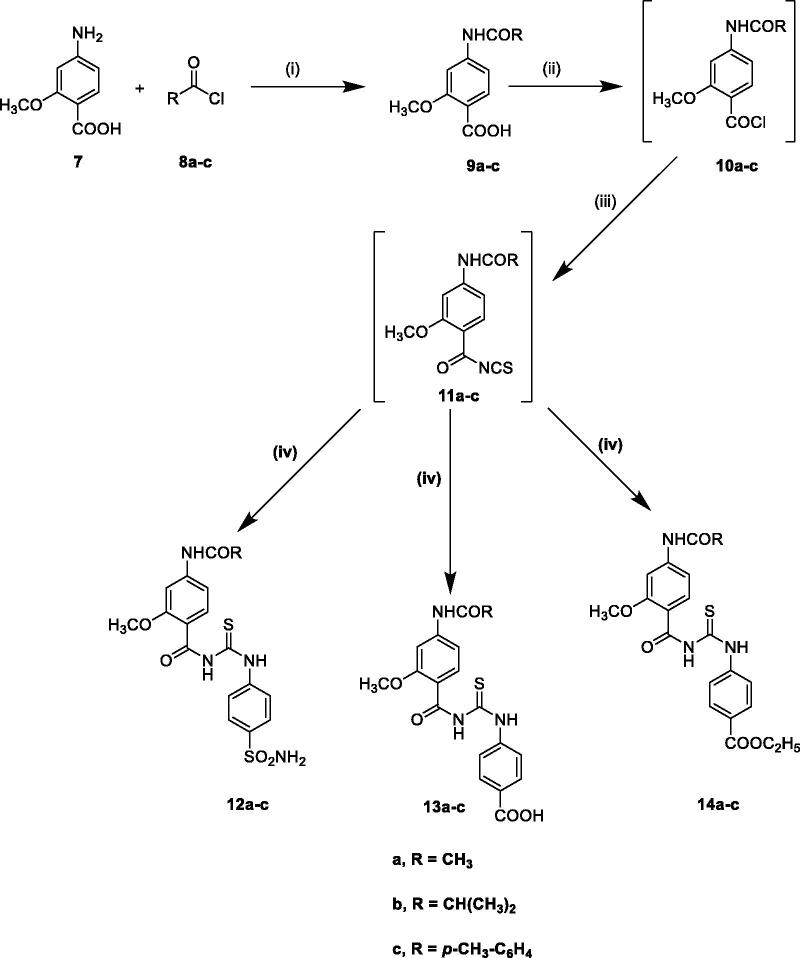
Reagents and reaction conditions: (i) KOH, acetonitrile, R.T, 1–2 h, (ii), SOCl_2_, methylene chloride, reflux, 4–5 h, (iii) NH_4_SCN, acetone, reflux, 1–3 h, (iv) sulphanilamide or 4-aminobenzoic acid or ethyl 4-aminobenzoate, acetone, reflux, 2–3 h.

The synthetic routes adopted for the synthesis of the target compounds **4a–d, 5a–d**, **6a–d, 12a–c, 13a–c,** and **14a–c** are depicted in Schemes 1 and 2, respectively.

The synthesis of compounds **4a–d**, **5a–d**, and **6a–d** was carried out by conversion of the appropriate benzoic acid derivative **1a–d** to the corresponding acid chloride **2a–d**, followed by reaction with ammonium thiocyanate to give the key intermediate benzoyl isothiocyanates **3a–d** that were treated similarly with sulphanilamide, 4-aminobenzoic acid or ethyl 4-aminobenzoate to furnish the target compounds (Scheme 1). IR spectra of compounds **4a–d** showed the appearance of characteristic bands at 3360–3255 cm^−1^corresponding to NH_2_ and NH, in addition to two stretching vibration bands at 1330–1160 cm^−1^ attributed to the characteristic SO_2_ group. ^1^H NMR spectra revealed the appearance of D_2_O exchangeable signal in the aromatic region around 7.42 ppm corresponding to two NH_2_ protons of the SO_2_NH_2_ group as a singlet.

IR spectrum of compound **5c** showed the appearance of characteristic carboxylic OH stretching vibration band at 3402 cm^−1^ and carbonyl band at 1654 cm^−1^. IR spectra of compounds **6a–d** displayed characteristic bands at 3380–3350 cm^−1^ corresponding to the NHs groups in addition to carbonyl bands of the ester at range of 1745–1712 cm^−1^. ^1^H NMR spectra of **6a–d** revealed the appearance of triplet signal at 1.30–1.42 ppm for protons of methyl group of esters (CH_3_CH_2_-) and quartette signal at 4.33 ppm corresponding to (CH_3_CH_2_-) protons. Further, ^13^C NMR spectra of **6b, 6c,** and **6d** displayed ethyl carbons CH_3_CH_2_ at 14.6 ppm, CH_3_CH_2_ carbon at 61.2 ppm along with carbonyl carbons at 165.7 ppm and CS carbon at 176.4–179.3 ppm.

Additionally, the synthesis of the target acetamido-benzoylureido derivatives **12a–c, 13a–c**, and **14a–c** was performed by acylation of 4-amino-2-methoxybenzoic acid **7** with different acyl chlorides **8a–c** in dry acetonitrile in the presence of potassium hydroxide to afford compounds **9a–c**. The key intermediates **10a–c** and **11a–c** were obtained *in situ* through the formation of the corresponding acid chlorides via the reaction of the acid derivatives with thionyl chloride followed by ammonium thiocyanate. The intermediates **11a–c** were not isolated but rather used in the synthesis of the target compounds by reaction with sulphanilamide, 4-aminobenzoic acid or ethyl 4-aminobenzoate in dry acetone to afford the target compounds **12a–c, 13a–c,** and **14a–c**, respectively in high yields (Scheme 2). The IR spectra of these compounds declared the presence of the expected new functional groups. The characteristics of the ^1^H NMR spectra of **10a–c** revealed the appearance of exchangeable NH protons at 7.27–7.41 ppm due to the sulphamoyl group, whereas compounds **11a–c** showed –COOH proton at 12.80–13.05 ppm. Also compounds **12a–c** displayed triplet-quartette signals for the ethyl protons at 1.32–1.43 and 4.32–4.46, respectively. This is in addition to the signals characteristic of the methyl, isopropyl, tolyl protons of the amide group of compounds **12a–c**, **13a–c,** and **14a–c**. On the other hand, ^13^C NMR spectra of **12a, 13a** and **14a** showed signal at 24.7 ppm corresponding to CH_3_ of acetamido group. Also, ^13^C NMR spectra of **12b** and **13b** revealed the isopropyl carbons (CHCH_3_)_2_ at 19.7 ppm and (CHCH_3_)_2_ carbon at 35.6 ppm. In addition, ^13^C NMR spectra of **13c** displayed a signal at 21.5 ppm attributed for CH_3_ carbons of the tolyl moiety.

### Biological evaluation

#### Carbonic anhydrase inhibitory activity

The CA inhibitory activities of all synthesised compounds **4a–d, 5a–d**, **6a–d, 12a–c, 13a–c,** and **14a–c**, as well as acetazolamide (**AAZ**) as a standard inhibitor were screened against four CA isoforms: hCA I, hCA II, hCA IX, and hCA XII. The selection of these four isoforms was based on the fact that hCA II is an antiglaucoma medication target, hCA IX and XII are well established targets for the therapy and prognosis of hypoxic malignancies, whereas, hCA I is one of the most common off-target isoforms for antiglaucoma and anticancer CAIs therapeutic application^22^.

The results showed that the inhibitory activity of the tested compounds against the four CA isoforms were highly dependent on the nature of the ZBG. In this context, the sulphamoyl derivatives (**4a–d** and **12a–c**) appeared as the most potent inhibitors among other derivatives, which revealed that the –COOH group failed to be a good ZBG. Interestingly, the ubiquitous cytosolic isoform hCA I, which is highly abundant in the gastrointestinal tract and red blood cells, was the most inhibited isoform among the others. The tested compound elicited mild activity against hCA II and hCA IX and a poor activity against hCA XII. A closer look on the results pointed out that seven compounds **4a, 4b, 4c, 4d, 6d**, **12a** and **12c** exhibited the best inhibitory activity against hCA I with K_I_ values ranging from 40.40–91.00 nM and better than the reference drug AAZ (250 nM). All of these compounds, except **6d**, featured a free sulphamoyl group which strongly supported the binding rational. Trying to figure out the effect of the tail substitution on the activity, it could be noticed that a small acetamido group as in compound **12a** was correlated to the highest activity against all hCA isoforms. Unsubstituted phenyl ring (compound **4a**) was associated with good to moderate activity, notably this compound was the most potent against hCA XII with *K*_I_ value = 57.40 nM. The presence of 3-chloro substitution (compound **4b**) gave the best activity agasinst hCA I, hCA II and h CA IX among the halogenated derivatives. The 3,4-dichloro and the 3-bromo derivatives, **4c** and **4d**, respectively, demonstarted good to moderate activity against hCA I, hCA II and h CA XII, where compound **4c** elicited the most potent inhibitory activity against hCA I (*K*_I_ = 40.40 nM). It is noteworthy that the 3-bromo ethylbenzoate derivative **6d** showed good to moderate activity against hCA I and hCA II.

Since the tested compounds were designed as modified derivatives of the lead compound SLC-0111, it deemed of interest to explore the impact of such modifications on the activity and selectivity of the targeted compounds. The results revealed a great increase in potency of the new compounds against hCA I and hCA II, especially compounds **4a–d**, **12a** and **12c** compared to SLC-0111. On the other hand, there was much reduction in potency against hCA IX and hCA XII in all compounds. Therefore, it could be claimed that the modifications performed on the structure of SLC-0111 in the present investigation led to a switch in selectivity of the compounds to the non-cancer related isoforms hCA I and hCA II.

## Conclusions

Twenty-one target compounds bearing a sulphamoyl, carboxylic or ethyl carboxylate substitutions and a diversely substituted phenyl tail moiety were designed as analogues of SLC-0111 and were synthesised through simple chemical procedures. All target compounds were assessed for their CA inhibitory activity against four relevant isoforms, namely hCA I, hCA II, hCA IX and hCA XII. The study revealed that the sulphamoyl group was the most efficient ZBG. Modifications of the substitution on the tail moiety had only a minor effect on activity and/or selectivity. Seven compounds **4a, 4b, 4c, 4d, 6d, 12a** and **12c** were selective against hCA I (Kis = 40.40–91.00 nM) compared to AAZ (Ki = 250.00 nM) which might present them as potential antiglaucoma drug candidates. Compounds bearing 4-acetamido-2-methoxy benzamido **12a** or 2-methoxy- (4-methylbenzamido) **12c** displayed superior activity (Kis= 67.60 and 91.00 nM) against hCAI more than that expressed by AAZ. Unlike SLC-0111, the targeted compounds were more selective to hCA I and hCA II rather than to hCA IX and hCA XII which would highlight these compounds as promising drug candidates for the treatment of glaucoma.

## Materials and methods

### Chemistry

#### General

All chemicals and solvents, which purchased and used without further purification. The melting points were measured using the SMP30 melting point apparatus. Thermo Scientific Nicolet iS10 spectrometer was used to record FT-IR spectra. ^1^HNMR spectra were run on a Bruker 400 MHz spectrophotometer. ^13^CNMR spectra were recorded in δ scale given in ppm on a Bruker 101 MHz spectrophotometer. Both types of spectra were performed in DMSO-*d_6._* Elemental analyses were performed on a Thermo Scientific Flash 2000 elemental analyser at the Regional Center for Mycology and Biotechnology, Al-Azhar University, Egypt. All reactions were monitored by silica gel 60 F254 TLC and visualised under UV light (254 nm). Compounds **2a–d**^24^, **3a–d**^25^, **4a,b**^26^, **5a**,**b,d, 6a**^27–29^, **9a–c**^24^, **10a–c**^25^, **11a–c**^26^ were prepared according to the reported procedures.

#### General procedure for the preparation of target compounds 4a–d, 5a–d, 6a–d, 12a–c, 13a–c and 14a–c

The freshly prepared benzoyl isothiocyanate derivative **3a–d** or **11a–c** (1 mmol) was treated with sulphanilamide, 4-aminobenzoic acid or ethyl 4-aminobenzoate (1 mmol) in refluxing anhydrous acetone (10 ml) for 2–3 h. The reaction mixture was cooled to room temperature and the formed precipitate was collected by filtration and recrystallized from ethanol to give the final target compounds in high yields^28^.

##### *N*-[(4-Sulphamoylphenyl)carbamothioyl]benzamide (4a)

Yellow crystals, (yield: 85%), m.p. 237–242 °C^30^.

##### 4-Chloro-*N*-[(4-sulfamoylphenyl)carbamothioyl]benzamide (4b)

Yellow crystals, (yield: 76%), m.p. 226–234 °C^30^.

##### 3,4-Dichloro-*N*-[(4-sulphamoylphenyl)carbamothioyl]benzamide (4c)

Yellow crystals, (yield: 75%), m.p. 206–208 °C; IR (KBr, *ν*_max_/cm^−1^): 3360, 3255 (NHs), 1670 (C=O), 1531 (C=S), 1327, 1157 (SO_2_); ^1^H NMR (DMSO-*d6*) *δ* ppm: 7.41 (s, 2H, NH_2,_ D_2_O exchangeable), 7.82 (d, 1H, *J* = 8.4 Hz, Ar-H), 7.85–7.94 (m, 5H, Ar-H), 8.25 (d, 1H, *J* = 2.0 Hz, Ar-H), 11.89 (s, 1H, NH, D_2_O exchangeable), 12.53 (s, 1H, NH, D_2_O exchangeable); MS (m/z): 404.20 [M]^+^, 406.47 [M + 2]^+^; Anal. Calcd. for C_14_H_11_Cl_2_N_3_O_3_S_2_ (404.28): C, 41.59; H, 2.74; N, 10.39; Found C, 41.78; H, 2.95; N, 10.57.

##### 4-Bromo-*N*-[(4-sulfamoylphenyl)carbamothioyl]benzamide (4d)

Yellow crystals, (yield: 75%), m.p. 215–217 °C; IR (KBr, *ν*_max_/cm^−1^): 3350–3245 (NHs), 1665 (C = O), 1520 (C = S), 1330, 1160 (SO_2_); ^1^H NMR *δ* ppm: 7.43 (s, 2H, NH_2,_ D_2_O exchangeable), 7.63 (d, 2H, *J* = 8.6 Hz, Ar-H), 7.83–7.87 (m, 4H, Ar-H), 8.02 (d, 2H, *J* = 8.6 Hz, Ar-H), 11.83 (s, 1H, NH, D_2_O exchangeable), 12.63 (s, 1H, NH, D_2_O exchangeable); Anal. Calcd. for C_14_H_12_BrN_3_O_3_S_2_ (414.29): C, 40.59; H, 2.92; N, 10.14; Found C, 40.47; H, 2.67; N, 10.03.

##### 4-(3-Benzoylthioureido)benzoic acid (5a)

Yellow crystals, (yield: 75%), m.p. 212–224 °C^31^.

##### 4-[3-(4-Chlorobenzoyl)thioureido]benzoic acid (5b)

Yellow crystals, (yield: 72%), m.p. 232–240 °C^31^.

##### 4-[3-(3,4-Dichlorobenzoyl)thioureido]benzoic acid (5c)

Yellow crystals, (yield: 67%), m.p. 216–218 °C; IR (KBr, ν_max_/cm^−1^): 3402 (OH), 3290 (br, NHs), 1678, 1654 (C = O), 1523 (C = S); ^1^H NMR *δ* ppm: 7.84–7.89 (m, 2H, Ar-H), 7.90–7.98 (m, 4H, Ar-H), 8.26 (s, 1H, Ar-H), 11.91 (s, 1H, NH, D_2_O exchangeable), 12.56 (s, 1H, NH, D_2_O exchangeable), 12.97 (s, 1H, OH, D_2_O exchangeable); Anal. Calcd. for C_15_H_10_Cl_2_N_2_O_3_S (369.22): C, 48.80; H, 2.73; N, 7.59; Found C, 49.07; H, 2.89; N, 7.80.

##### 4-[3-(4-Bromobenzoyl)thioureido]benzoic acid (5d)

Yellow crystals, (yield: 60%), m.p. 206–215 °C^31^.

##### Ethyl-4-(3-benzoylthioureido)benzoate (6a)

Yellow crystals, (yield: 67%), m.p. 223–235 °C^32^.

##### Ethyl 4-[3-(4-chlorobenzoyl)thioureido]benzoate (6b)

Yellow crystals, (yield: 75%), m.p. 219–221 °C; IR (KBr, ν_max_/cm^−1^): 3350–3360 (NHs), 1740, (C = O) ester, 1665, (C = O) amide, 1535 (C = S); ^1^H NMR *δ* ppm: 1.42 (t, 3H, *J* = 1.6 Hz, CH_3_), 4.33 (q, 2H, *J* = 6.0 Hz, CH_2_), 7.29–7.36 (m, 4H, Ar-H), 7.94–8.00 (m, 4H, Ar-H), 8.97 (s, 2H, 2NH, D_2_O exchangeable); ^13^C NMR *δ* ppm: 14.6, 61.3, 110.3, 129.4, 129.7, 130.02, 130.08, 131.3, 142.9, 145.9, 165.7, 176.4; Anal. Calcd. for C_17_H_15_ClN_2_O_3_S (362.83): C, 56.28; H, 4.17; N, 7.72; Found C, 56.43; H, 4.28; N, 7.94.

##### Ethyl 4-[3-(3,4-dichlorobenzoyl)thioureido]benzoate (6c)

Yellow crystals, (yield: 67%), m.p. 216–218 °C; IR (KBr, ν_max_/cm^−1^): 3186 (NHs), 1712, 1693 (C = O), 1535 (C = S); ^1^H NMR *δ* ppm: 1.33 (t, 3H, *J* = 7.1 Hz, CH_3_), 4.33 (q, 2H, *J* = 10.0 Hz, CH_2_), 7.34 (d, 2H, *J* = 8.4 Hz, Ar-H), 7.48 (d, 2H, *J* = 8.6 Hz, Ar-H), 7.90–8.02 (m, 4H, Ar-H), 8.95 (s, 1H, Ar-H), 11.87 (s, 1H, NH, D_2_O exchangeable), 12.58 (s, 1H, NH, D_2_O exchangeable); ^13^C NMR *δ* ppm: 14.6, 61.2, 110.3, 124.0, 129.4, 129.7, 130.2, 131.7, 133.0, 136.3, 142.6, 145.9, 165.7, 176.4, 179.2; MS (*m*/*z*): 397.27 [M]^+^, 396.68 [M]^+^, 399.34 [M + 2]^+^; Anal. Calcd. for C_17_H_14_Cl_2_N_2_O_3_S (397.27): C, 51.40; H, 3.55; N, 7.05; Found C, 51.67; H, 3.62; N, 7.19.

##### Ethyl-4-[3-(4-bromobenzoyl)thioureido]benzoate (6d)

Yellow crystals, (yield: 75%), m.p. 218–220 °C; IR (KBr, *ν*_max_/cm^−1^): 3360–3380 (NHs), 1745, (C = O) ester, 1660, (C = O) amide, 1535 (C = S); ^1^H NMR *δ* ppm: 1.33 (t, 3H, *J* = 7.0 Hz, CH_3_), 4.32 (q, 2H, *J* = 7.0 Hz, CH_2_), 7.76 (d, 2H, *J* = 8.5 Hz, Ar-H), 7.91 (d, 4H, *J* = 8.6 Hz, Ar-H), 8.00 (d, 2H, *J* = 8.6 Hz, Ar-H), 11.77 (s, 1H, NH, D_2_O exchangeable), 12.69 (s, 1H, NH, D_2_O exchangeable); ^13^C NMR *δ* ppm: 14.6, 61.2, 123.9, 127.5, 127.6, 130.2, 131.2, 131.7, 131.9, 142.6, 165.5, 167.7, 179.3; Anal. Calcd. for C_17_H_15_BrN_2_O_3_S (407.28): C, 50.13; H, 3.71; N, 6.88; Found C, 50.39; H, 3.85; N, 7.12.

##### 4-Acetamido-2-methoxy-*N*-[(4-sulfamoylphenyl)carbamothioyl]benzamide (12a)

Yellow crystals, (yield: 75%), m.p. 225–227 °C; IR (KBr, *ν*_max_/cm^−1^): 3340, 3302 (NHs), 1697, 1666 (C = O), 1519 (C = S), 1338, 1157 (SO_2_); ^1^H NMR *δ* ppm: 2.11 (s, 3H, CH_3_), 3.90 (s, 3H, OCH_3_), 7.35 (d, *J* = 8, 1H, Ar-H), 7.41 (s, 2H, NH_2,_D_2_O exchangeable), 7.65–7.87 (m, 3H, Ar-H), 7.95–7.98 (m, 3H, Ar-H), 10.41 (s, 1H, NH, D_2_O exchangeable), 11.12 (s, 1H, NH, D_2_O exchangeable), 12.78 (s, 1H, NH, D_2_O exchangeable); ^13^C NMR *δ* ppm: 24.7, 57.0, 102.5, 112.1, 113.2, 124.6, 126.7, 133.0, 141.1, 141.8, 146.3, 159.0, 164.8, 169.8; MS (*m*/*z*): 422.66 [M]^+^; Anal. Calcd. for C_17_H_18_N_4_O_5_S_2_ (422.47): C, 48.33; H, 4.29; N, 13.26; Found C, 48.61; H, 4.45; N, 13.50.

##### 4-Isobutyramido-2-methoxy-*N*-[(4-sulfamoylphenyl)carbamothioyl] benzamide (12b)

White crystals, (yield: 79%), m.p. 248–250 °C; IR (KBr, *ν*_max_/cm^−1^): 3300–3310 (NHs), 1710, 1690, (C = O) amide, 1520 (C = S), 1336, 1135 (SO_2_); ^1^H NMR *δ* ppm: 1.13 (d, 6H, *J* = 6.8 Hz,-CH(CH_3_)_2_), 2.61–2.68 (m, 1H, -CH(CH_3_)_2_), 4.01 (s, 3H, OCH_3_), 7.36 (d, 1H, *J* = 7.1 Hz, Ar-H), 7.40 (s, 2H, NH_2_, D_2_O exchangeable), 7.73 (s, 1H, Ar-H), 7.86 (d, 2H, *J* = 8.6 Hz, Ar-H), 7.92–7.99 (m, 3H, Ar-H), 10.32 (s, 1H, NH, D_2_O exchangeable), 11.11 (s, 1H, NH, D_2_O exchangeable), 12.77 (s, 1H, NH, D_2_O exchangeable); ^13^C NMR *δ* ppm: 19.7, 35.6, 57.0, 102.6, 112.2, 113.1, 124.6, 126.7, 133.0, 141.1, 141.9, 146.5, 159.0, 164.7, 176.6, 178.7; Anal. Calcd. for C_19_H_22_N_4_O_5_S_2_ (450.53): C, 50.65; H, 4.92; N, 12.44; Found C, 50.89; H, 4.81; N, 12.67.

##### 2-Methoxy-4-(4-methylbenzamido)-*N*-[(4-sulfamoylphenyl)carbamothioyl] benzamide (12c)

Yellow crystals, (yield: 82%), m.p. 250–252 °C; IR (KBr, *ν*_max_/cm^−1^): 3350–3205 (NHs), 1680, 1650, (C = O) amide, 1530 (C = S), 1320, 1154 (SO_2_); ^1^H NMR *δ* ppm: 2.40 (s, 3H, CH_3_), 3.82 (s, 3H, OCH_3_), 7.27 (s, 2H, NH_2,_ D_2_O exchangeable), 7.36 (d, 2H, *J* = 7.9, Ar-H), 7.40 (d, 1H, *J* = 8.4, Ar-H), 7.46–7.50 (m, 1H, Ar-H), 7.69–7.72 (m, 1H, Ar-H), 7.80 (d, 2H, *J* = 8.8, Ar-H), 7.90 (d, 2H, *J* = 8.0, Ar-H), 7.96 (d, 2H, *J* = 8.6, Ar-H), 10.38 (s, 1H, NH, D_2_O exchangeable), 10.48 (s, 2H, 2NH, D_2_O exchangeable); Anal. Calcd. for C_23_H_22_N_4_O_5_S_2_ (498.57): C, 55.41; H, 4.45; N, 11.24; Found C, 55.63; H, 4.61; N, 11.48.

##### 4-[3-(4-Acetamido-2-methoxybenzoyl)thioureido]benzoic acid (13a)

Brown crystals, (yield: 82%), m.p. 245–247 °C; ^1^H NMR *δ* ppm: 2.10 (s, 3H, CH_3_), 4.00 (s, 3H, OCH_3_), 7.34 (d, 1H, *J* = 7.1, Ar-H), 7.64 (s, 1H, Ar-H), 7.90–8.00 (m, 5H, Ar-H), 10.42 (s, 1H, NH, D_2_O exchangeable), 11.11 (s, 1H, NH, D_2_O exchangeable), 12.85 (s, 1H, NH, D_2_O exchangeable), 13.05 (s, 1H, OH, D_2_O exchangeable). ^13^C NMR *δ* ppm: 24.7, 57.0, 102.5, 112.0, 113.1, 123.7, 128.6, 130.4, 131.5, 133.0, 142.1, 146.3, 159.0, 164.8, 167.1, 169.8, 178.2; Anal. Calcd. for C_18_H_17_N_3_O_5_S (387.41): C, 55.81; H, 4.42; N, 10.85; Found C, 56.08; H, 4.56; N, 11.07.

##### 4-[3-(4-Isobutyramido-2-methoxybenzoyl)thioureid])benzoic acid (13b)

Brown crystals, (yield: 76%), m.p. 256–258 °C; IR (KBr, *ν*_max_/cm^−1^): 3313 (br NHs, OH), 1689, 1670 (C = O), 1516 (C = S); ^1^H NMR *δ* ppm: 1.12 (d, 6H, *J* = 6.8 Hz, CH(CH_3_)_2_), 2.60–2.67 (m, 1H, CH(CH_3_)_2_), 4.00 (s, 3H, OCH_3_), 7.35 (d, 1H, *J* = 7.5 Hz, Ar-H), 7.72 (s, 1H, Ar-H), 7.92 (t, 3H, *J* = 8.2 Hz, Ar-H), 7.97 (t, 2H, *J* = 6.7 Hz, Ar-H), 10.30 (s, 1H, NH, D_2_O exchangeable), 11.09 (s, 1H, NH, D_2_O exchangeable), 12.86 (s, 1H, NH, D_2_O exchangeable), 12.97 (s, 1H, OH, D_2_O exchangeable). ^13^C NMR *δ* ppm: 19.7, 35.6, 56.9, 102.5, 112.1, 112.7, 123.4, 128.5, 130.4, 133.0, 142.0, 146.6, 158.9, 164.6, 167.1, 176.6, 178.1; MS (*m*/*z*): 416.32 [M + 1]^+^; Anal. Calcd. For C_20_H_21_N_3_O_5_S (415.46): C, 57.82; H, 5.10; N, 10.11; Found C, 57.95; H, 5.23; N, 10.40.

##### 4-(3-(2-Methoxy-4-(4-methylbenzamido)benzoyl)thioureido]benzoic acid (13c)

Yellow crystals, (yield: 78%), m.p. 281–283 °C; IR (KBr, *ν*_max_/cm^−1^): 3353 (NHs), 3286 (OH) acide, 1687 (C = O) acide, 1670, 1650 (C = O) amide, 1553 (C = S); ^1^H NMR *δ* ppm: 2.09 (s, 3H, CH_3_), 4.05 (s, 3H, OCH_3_), 7.36 (d, 2H, *J* = 10.0 Hz, Ar-H), 7.67–7.95 (m, 7H, Ar-H), 8.00 (d, 2H, *J* = 8.6 Hz, Ar-H), 10.46 (s, 1H, NH, D_2_O exchangeable), 10.85 (s, 1H, NH, D_2_O exchangeable), 11.15 (s, 1H, NH, D_2_O exchangeable), 12.80 (s, 1H, OH, D_2_O exchangeable). ^13^C NMR *δ* ppm: 21.5, 57.1, 113.6, 119.9, 123.7, 125.8, 128.6, 129.4, 129.5, 130.4, 130.6, 132.1, 142.4, 143.8, 146.4, 158.8, 164.8, 166.2, 167.4, 178.3; Anal. Calcd. for C_24_H_21_N_3_O_5_S (463.51): C, 62.19; H, 4.57; N, 9.07; Found C, 61.97; H, 4.73; N, 9.34.

##### Ethyl-4-[3-(4-acetamido-2-methoxybenzoyl)thioureido]benzoate (14a)

Brown crystals, (yield: 68%), m.p. 237–239 °C; IR (KBr, *ν*_max_/cm^−1^): 3487, 3205 (NHs), 1700 (C = O) ester, 1675, 1635 (C = O) amide, 1531 (C = S); ^1^H NMR *δ* ppm: 1.32 (t, 3H*, J* = 7.1 Hz, CH_2_CH_3_), 2.09 (s, 3H, CH_3_), 4.01 (s, 3H, OCH_3_), 4.33 (q, 2H, *J* = 7.1 Hz, CH_2_CH_3_), 7.34 (d, 1H, *J* = 8.6, Ar-H), 7.65 (s, 1H, Ar-H), 7.96 (d, *J* = 8.5 Hz, 3H, Ar-H), 7.98–8.02 (m, 2H, Ar-H), 10.42 (s, 1H, NH, D_2_O exchangeable), 11.12 (s, 1H, NH, D_2_O exchangeable), 12.88 (s, 1H, NH, D_2_O exchangeable); ^13^C NMR *δ* ppm: *δ* 14.6, 24.7, 57.0, 61.2, 102.5, 112.0, 113.1, 123.8, 127.6, 130.2, 133.0, 142.4, 146.3, 159.0, 164.8, 165.5, 169.8, 178.3; Anal. Calcd. for C_20_H_21_N_3_O_5_S (415.46): C, 57.82; H, 5.10; N, 10.11; Found C, 58.04; H, 5.19; N, 10.37.

##### Ethyl-4-[3-(4-isobutyramido-2-methoxybenzoyl)thioureido]benzoate (14b)

White crystals, (yield: 75%), m.p. 245–247 °C; IR (KBr, *ν*_max_/cm^−1^): 3302, 3186 (NHs), 1712, 1693,1666 (C = O), 1512 (C = S); ^1^H NMR *δ* ppm: 1.13 (d, *J* = 6.8 Hz, 6H, CH(CH_3_)_2_), 1.33 (t, 3H*, J* = 7.1 Hz, -CH_2_CH_3_), 2.58–2.67 (m, 1H, CH(CH_3_)_2_), 4.01 (s, 3H, OCH_3_), 4.32 (q, 2H, *J* = 4.1 Hz, -CH_2_CH_3_), 7.33–7.37 (m, 2H, Ar-H), 7.73 (s, 1H, Ar-H), 7.93–8.01 (m, 4H, Ar-H), 10.31 (s, 1H, NH, D_2_O exchangeable), 11.10 (s, 1H, NH, D_2_O exchangeable), 12.88 (s, 1H, NH, D_2_O exchangeable); ^13^C NMR *δ* ppm: 14.6, 19.7, 31.0, 57.0, 61.3, 102.6, 110.7, 112.2, 123.6, 127.6, 129.4, 133.0, 142.4, 146.5, 159.0, 164.7, 165.5, 165.7, 178.2. MS (*m*/*z*): 444.25 [M + 1]^+^; Anal. Calcd. for C_22_H_25_N_3_O_5_S (443.52): C, 59.58; H, 5.68; N, 9.47; Found C, 59.79; H, 5.80; N, 9.71.

##### Ethyl-4-[3-(2-methoxy-4-(4-methylbenzamido)benzoyl)thioureido]benzoate (14c)

White crystals, (yield: 80%), m.p. 257–259 °C; IR (KBr, *ν*_max_/cm^−1^): 3205 (br, NHs), 1712 1693,1633 (C = O), 1531 (C = S); ^1^H NMR *δ* ppm: 1.43 (t, 3H, *J* = 6.3 Hz, CH_2_CH_3_), 2.44 (s, 3H, CH_3_), 4.02 (s, 3H, OCH_3_), 4.46 (q, 2H, *J* = 7.0 Hz, -CH_2_CH_3_), 7.38 (s, 1H, Ar-H), 7.55 (d, *J* = 7.9 Hz, 2H, Ar-H), 7.71 (d, *J* = 7.4 Hz, 1H, Ar-H), 8.01–8.08 (m, 7H, Ar-H), 10.57 (s, 1H, s, 1H, NH. D_2_O exchangeable), 11.82 (s, 1H, NH, D_2_O exchangeable), 12.90 (s, 1H, NH, D_2_O exchangeable); Anal. Calcd. for C_26_H_25_N_3_O_5_S (491.56): C, 63.53; H, 5.13; N, 8.55; Found C, 63.39; H, 5.31; N, 8.79.

### Biological evaluation

#### Carbonic anhydrase inhibitory activity

A stopped flow CO_2_ hydrase assay was adopted using an SX.18 MV-R Applied Photophysics (Oxford, UK) stopped-flow instrument to assess the inhibition against the various CA isozymes^33^. Phenol red(at a concentration of 0.2 mM has been used as an indicator, working at the absorbance maximum of 557 nm, with 20 Mm Hepes (pH 7.5) as buffer, and 20 mM Na_2_SO_4._ The initial rates of the CA-catalysed CO_2_ hydration reaction was run for a period of 10–100 s then completing as the reported protocol. The inhibition constants were obtained by non-linear least-squares methods using PRISM 3 and the Cheng–Prusoff equation, and represent the mean for at least three different determinations^34–36^. All CA isofoms were recombinant ones obtained in-house as previously reported^37–42^.

## Supplementary Material

Supplemental Material
