# Supplementary material for: Design and synthesis of some new benzoylthioureido phenyl derivatives targeting carbonic anhydrase enzymes
Source: J Enzyme Inhib Med Chem. 2022 Sep 27;37(1):2702–9. doi: 10.1080/14756366.2022.2126463 (PMC9542353; doi:10.1080/14756366.2022.2126463)
Supplement: Supplemental Material [file IENZ_A_2126463_SM6461.pdf]

Design and synthesis of some new benzoylthioureido phenyl derivatives targeting carbonic anhydrase enzymes

Mazin A.A. Najm[a] , Azza T. Shalaby[b,c] , Safinaz E-S Abbas[d] , Fadi M. Awadallah[d] , Heba. A. Allam[d] , Daniela Vullo[e]* , Claudiu T. Supuran[e] ,Walaa R. Mahmoud[d*]

[a] *Pharmaceutical Chemistry Department, College of Pharmacy, Al-Ayen University, Thi-Qar, Iraq*

[b] *Pharmaceutical Organic Chemistry, Faculty of Pharmacy, Cairo University*

c Pharmaceutical Organic Chemistry, Faculty of Pharmacy, October 6 university

d Pharmaceutical Chemistry Department, Faculty of Pharmacy, Cairo University, Kasr El-Eini Street, 11562, Cairo, Egypt

e Department of NEUROFARBA, Section of Pharmaceutical and Nutraceutical Sciences, University of Florence, Polo Scientifico, Via U. Schiff 6, 50019, Sesto Fiorentino, Firenze, Italy.

Information Classification: General

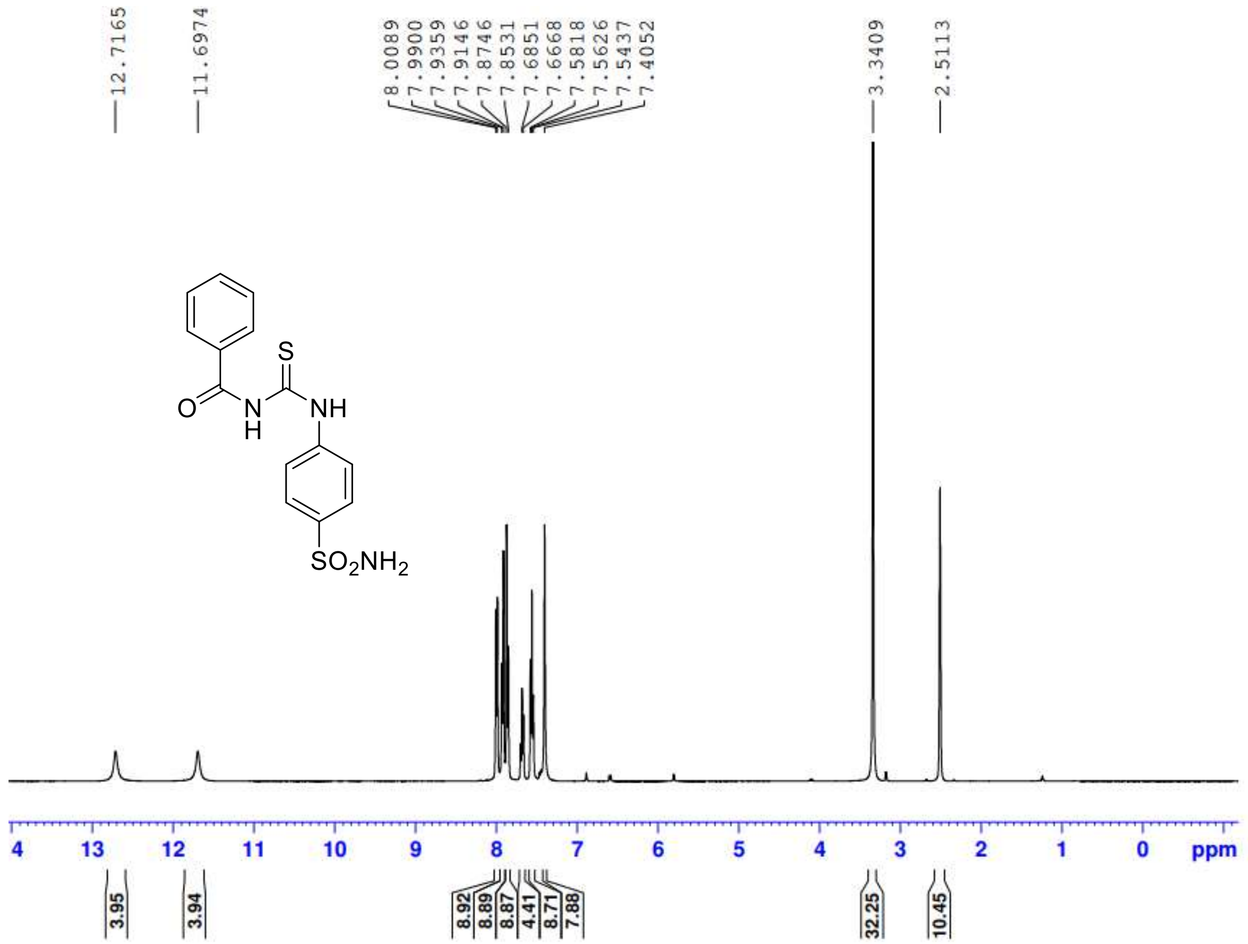

Figure 1: ^{1}H-NMR spectrum of N-[(4-Sulfamoylphenyl)carbamothioyl]benzamide (**4a**)

Information Classification: General

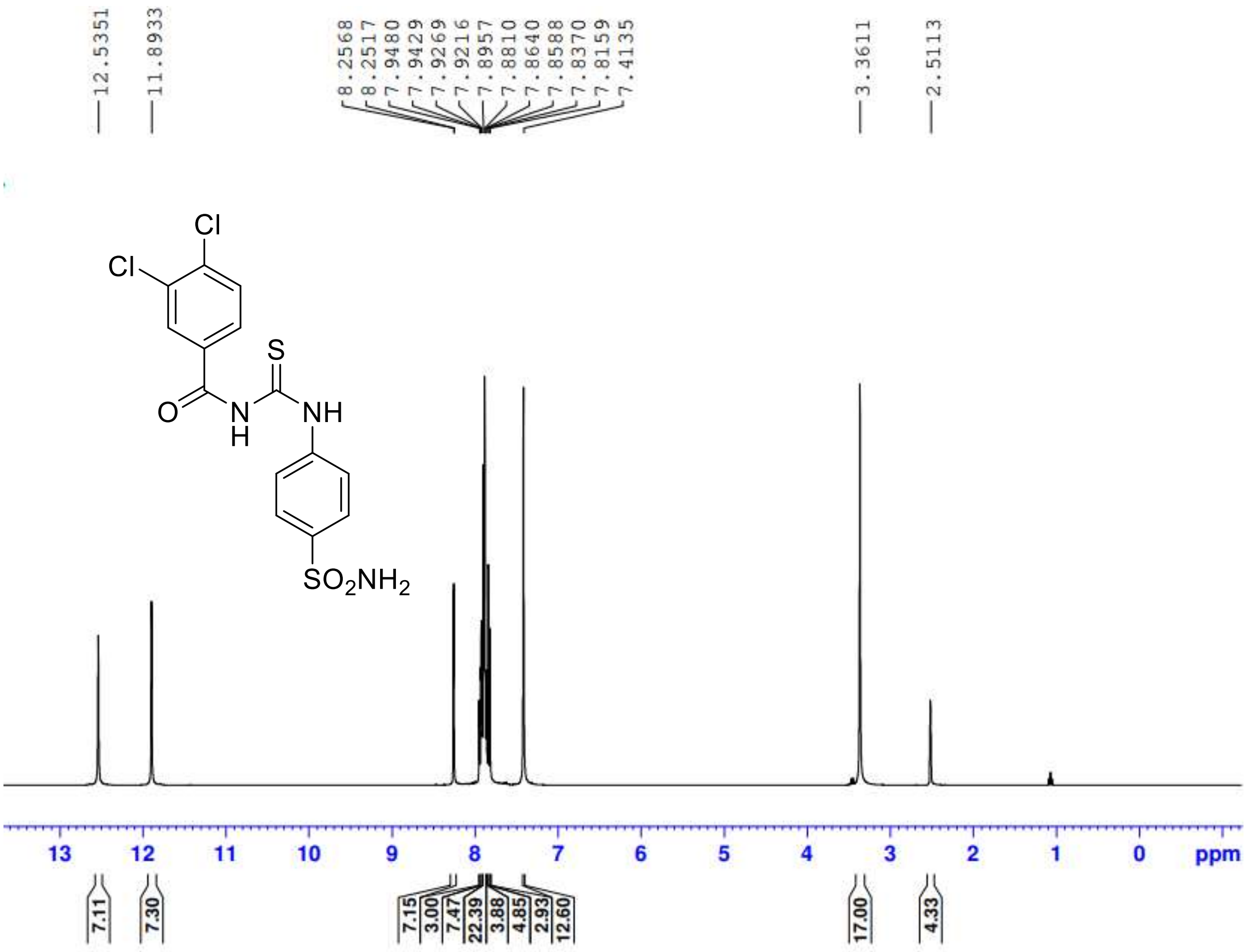

Figure 2: ^{1}H-NMR spectrum of 3,4-Dichloro-N-[(4-sulfamoylphenyl)carbamothioyl]benzamide (**4c**)

Information Classification: General

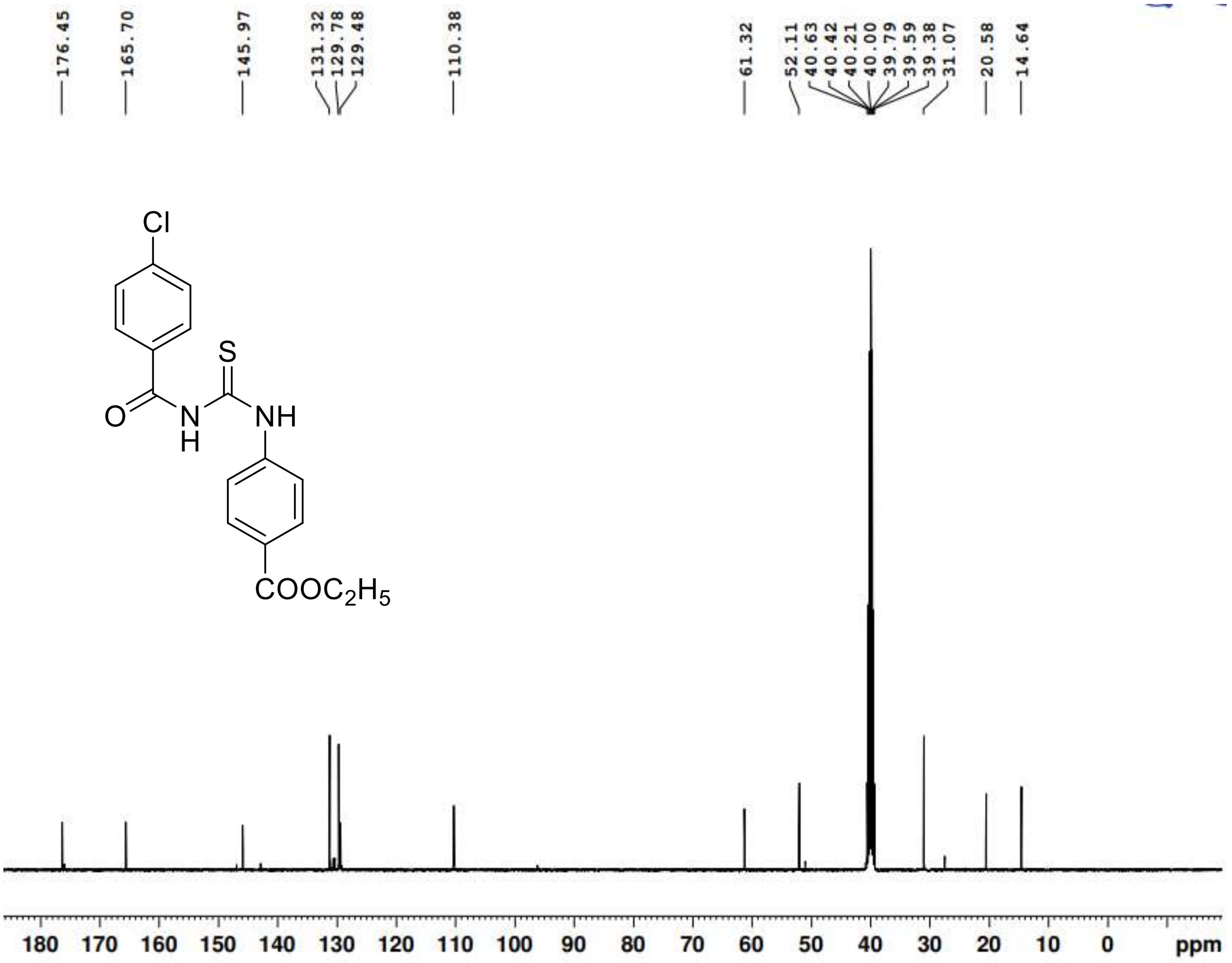

Figure 3: ^{13}C-NMR spectrum of ethyl 4-[3-(4-chlorobenzoyl)thioureido]benzoate **(6b)**

Information Classification: General

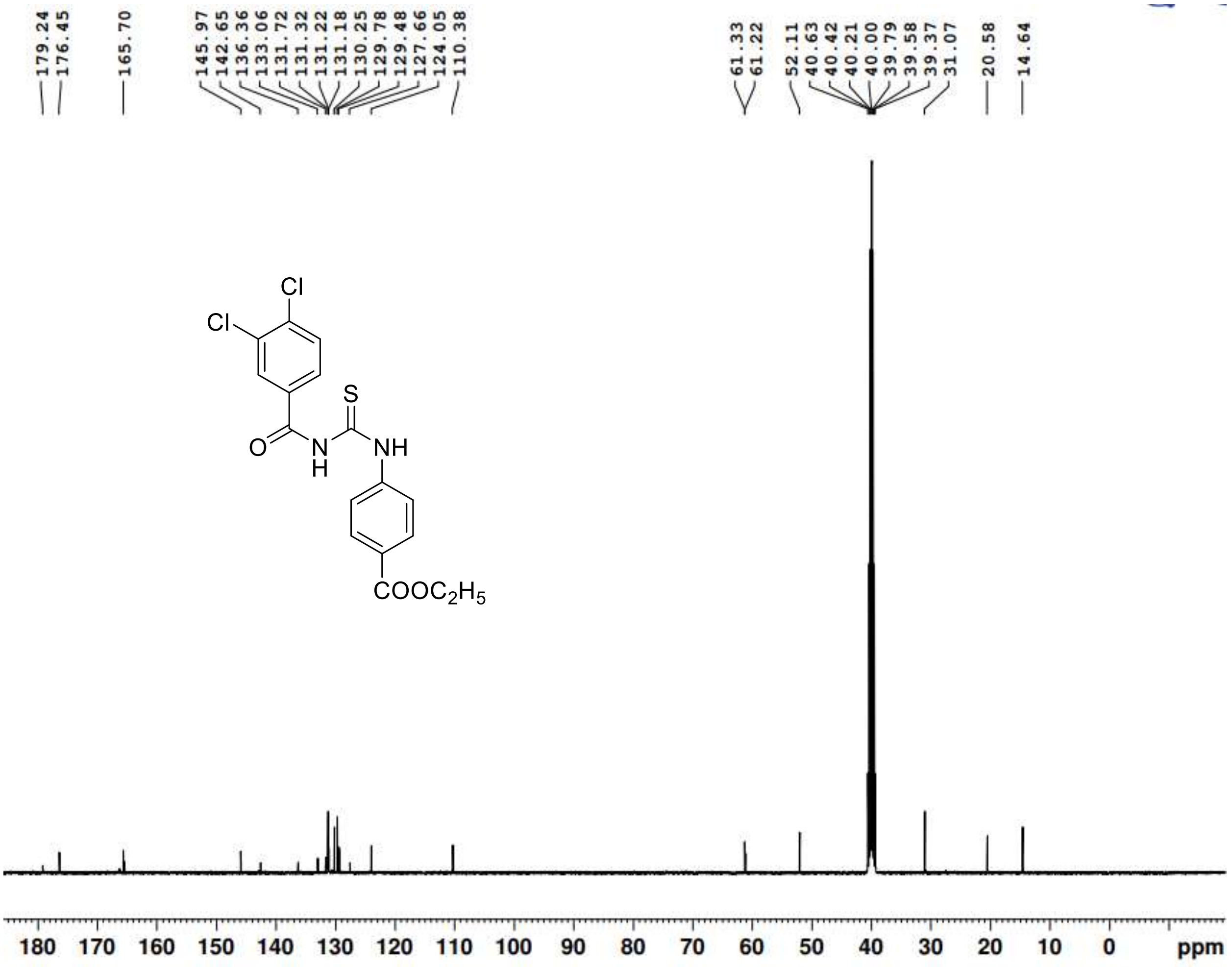

Figure 4: ^{13}C-NMR spectrum of Ethyl 4-[3-(3,4-dichlorobenzoyl)thioureido]benzoate **(6c)**

Information Classification: General

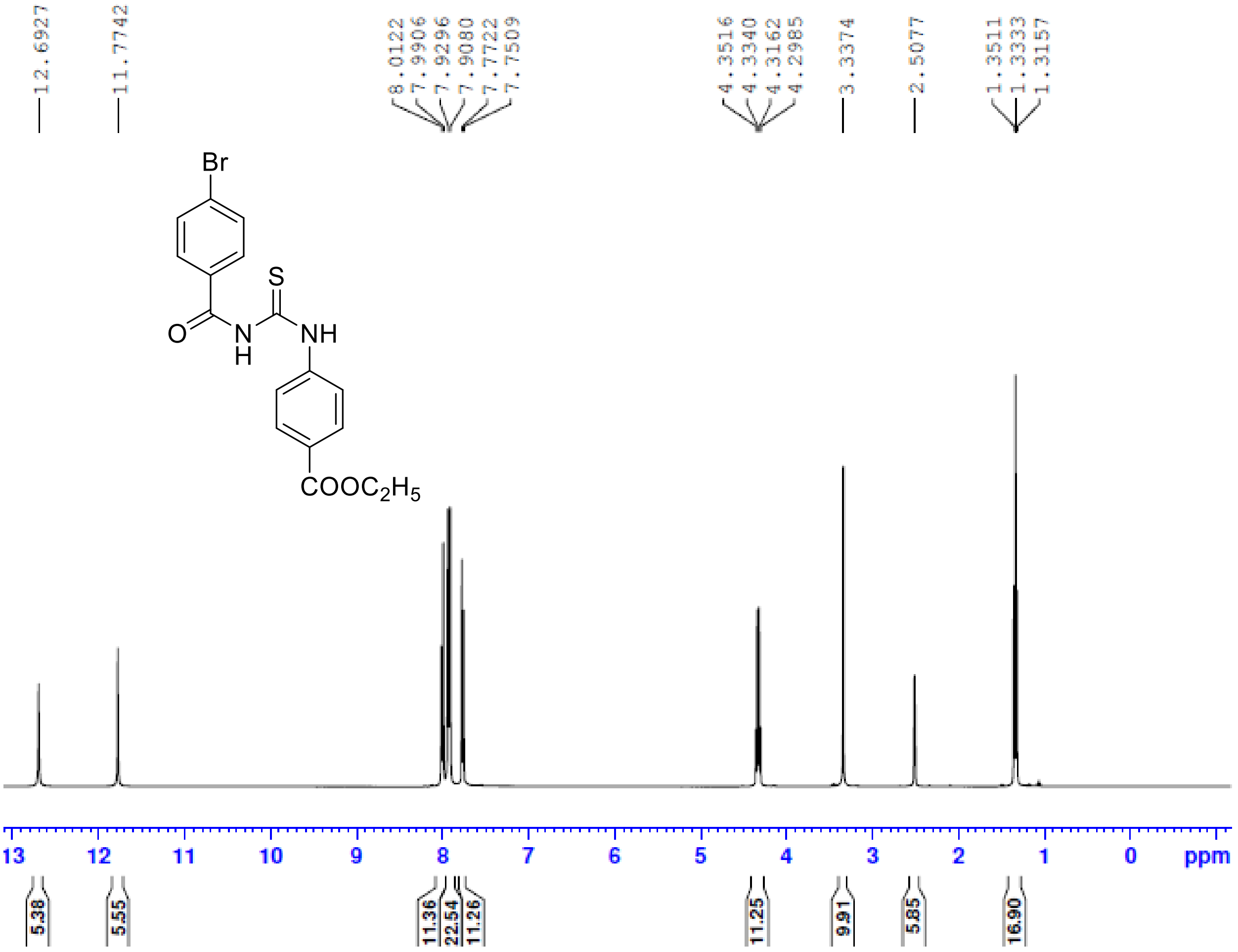

Figure 5: ^{1}H-NMR spectrum of Ethyl-4-[3-(4-bromobenzoyl)thioureido]benzoate **(6d)**

Information Classification: General

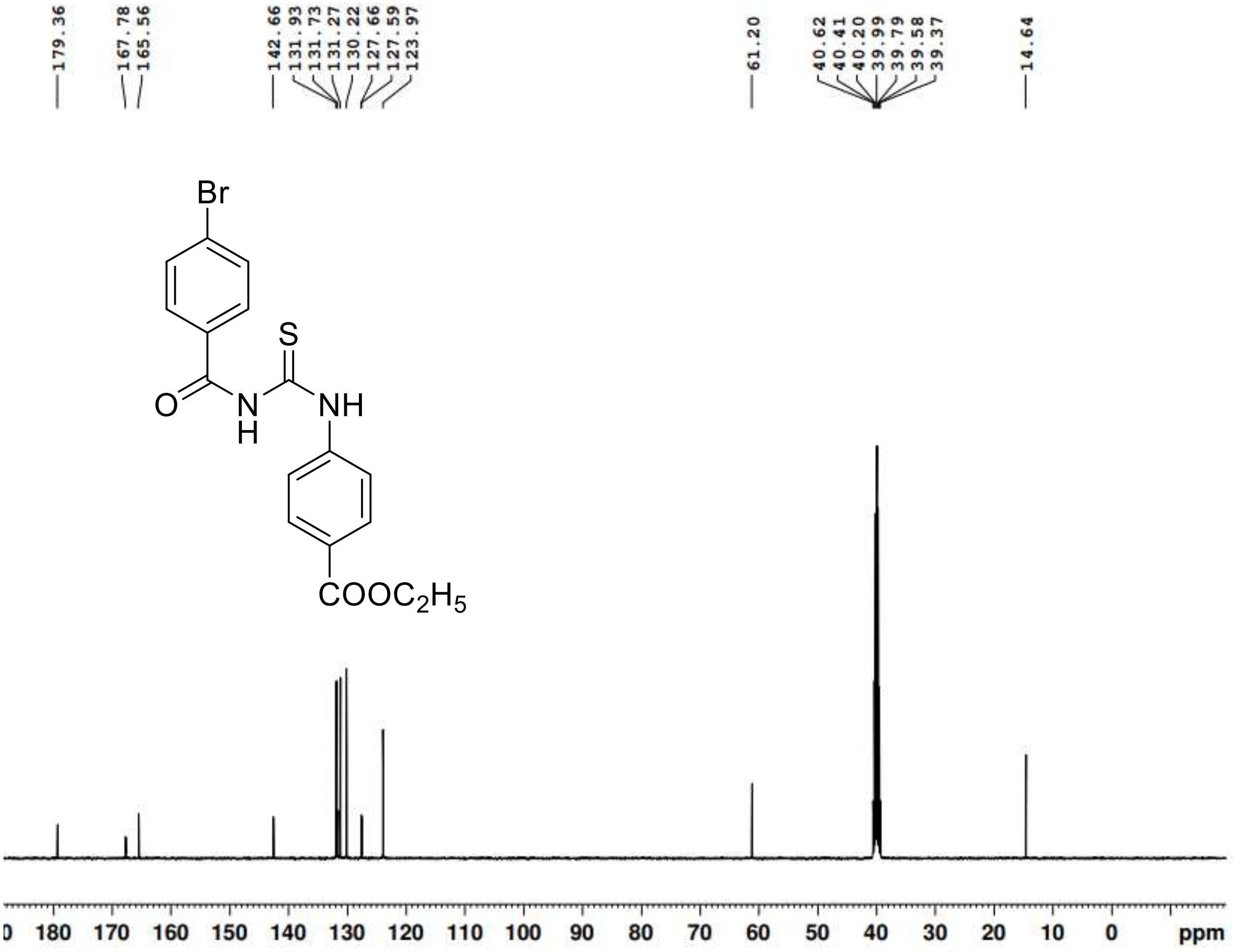

Figure 6: ^{13}C-NMR spectrum of ethyl-4-[3-(4-bromobenzoyl)thioureido]benzoate **(6d)**

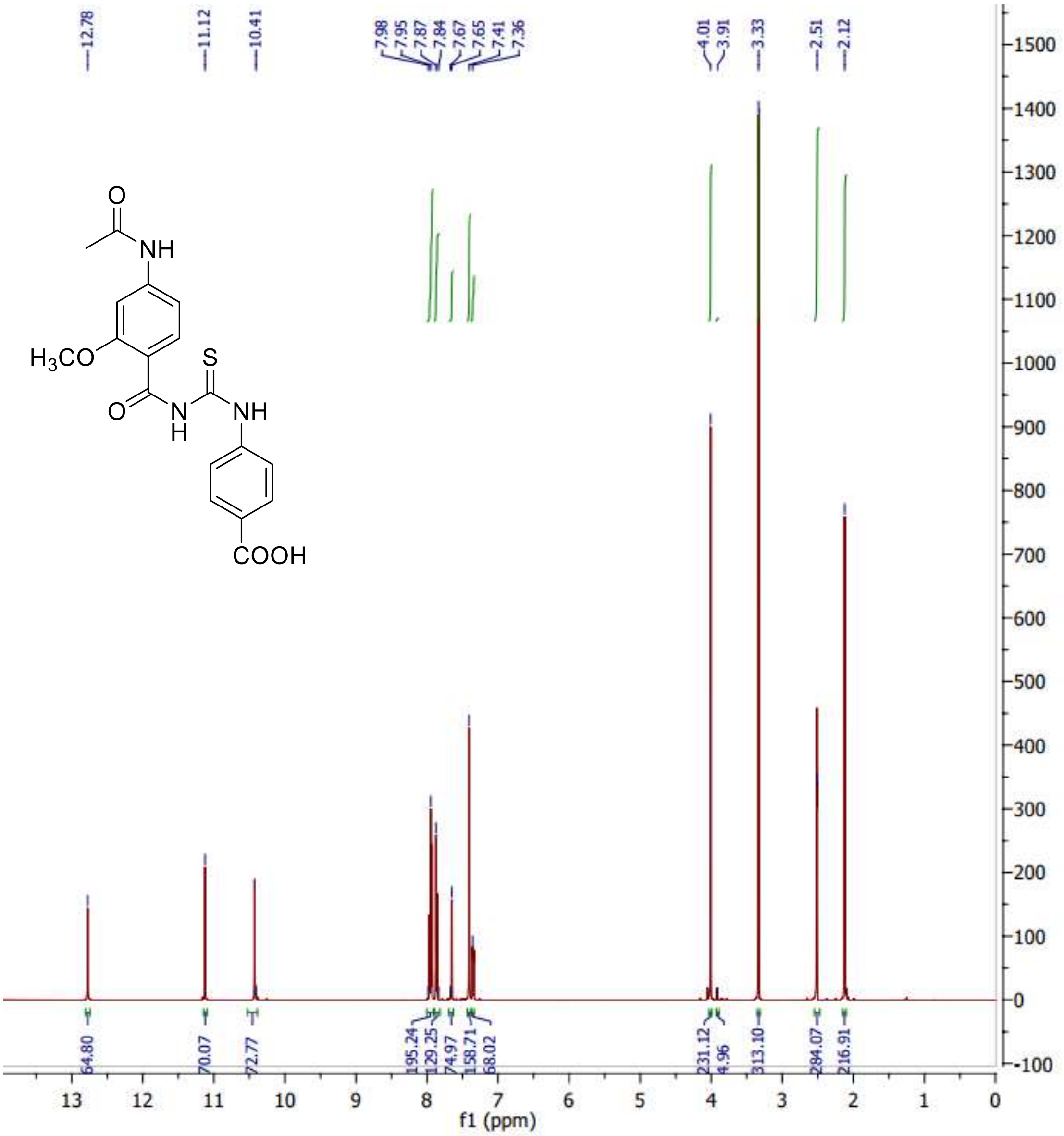

Figure 7: ^{1}H-NMR spectrum of 4-Acetamido-2-methoxy-N-[(4-sulfamoylphenyl) carbamothioyl]benzamide (**12a**)

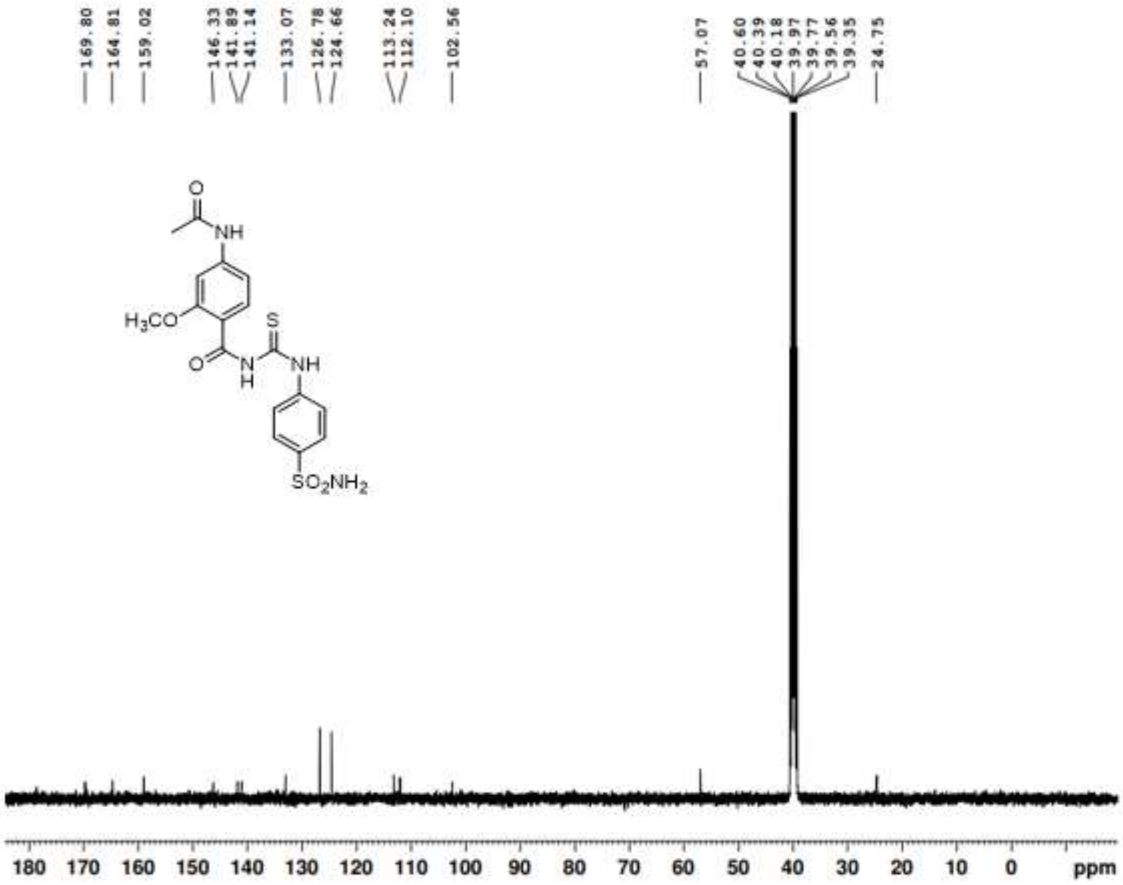

Figure 8: ^{13}C-NMR spectrum of 4-Acetamido-2-methoxy-N-[(4-sulfamoyl phenyl)carbamothioyl]benzamide **(12a)**

Information Classification: General

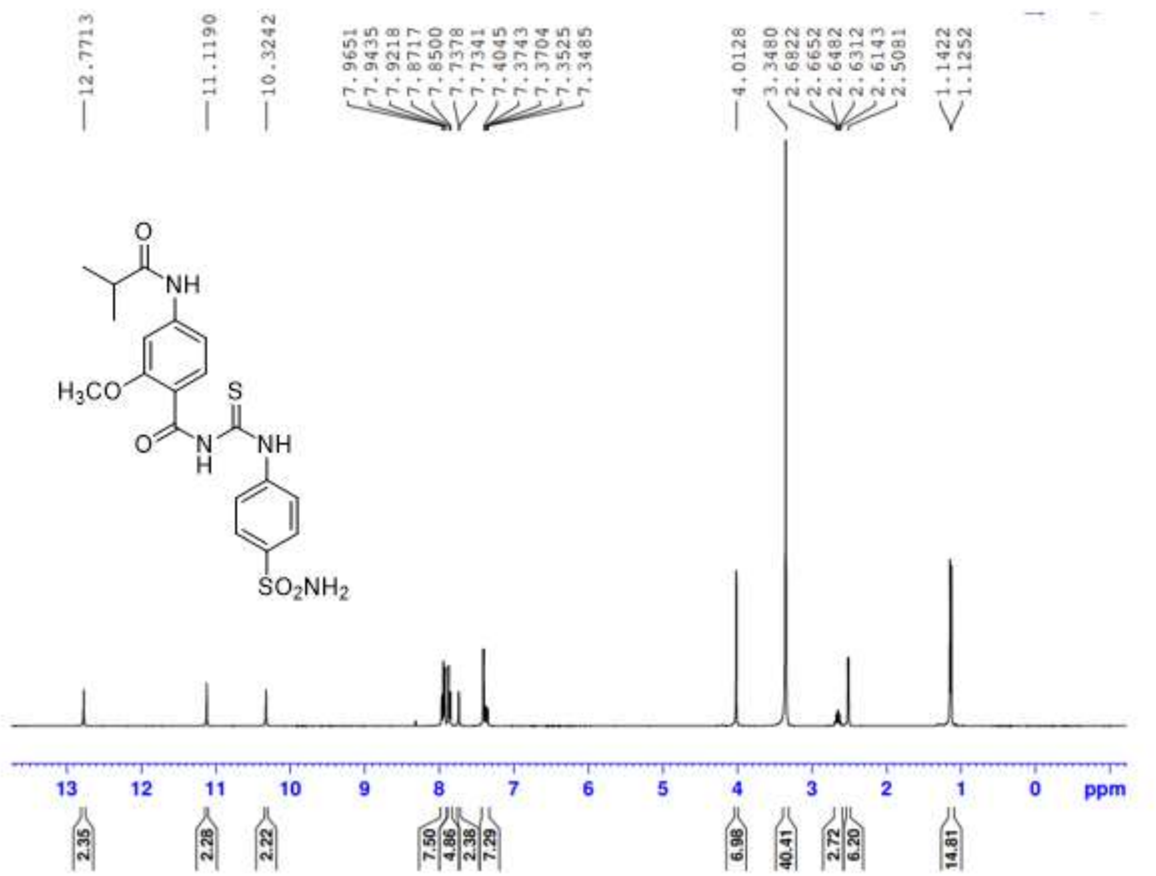

Figure 9: 4-Isobutyramido-2-methoxy-*N*-[(4-sulfamoylphenyl)carbamothioyl] benzamide **(12b)**

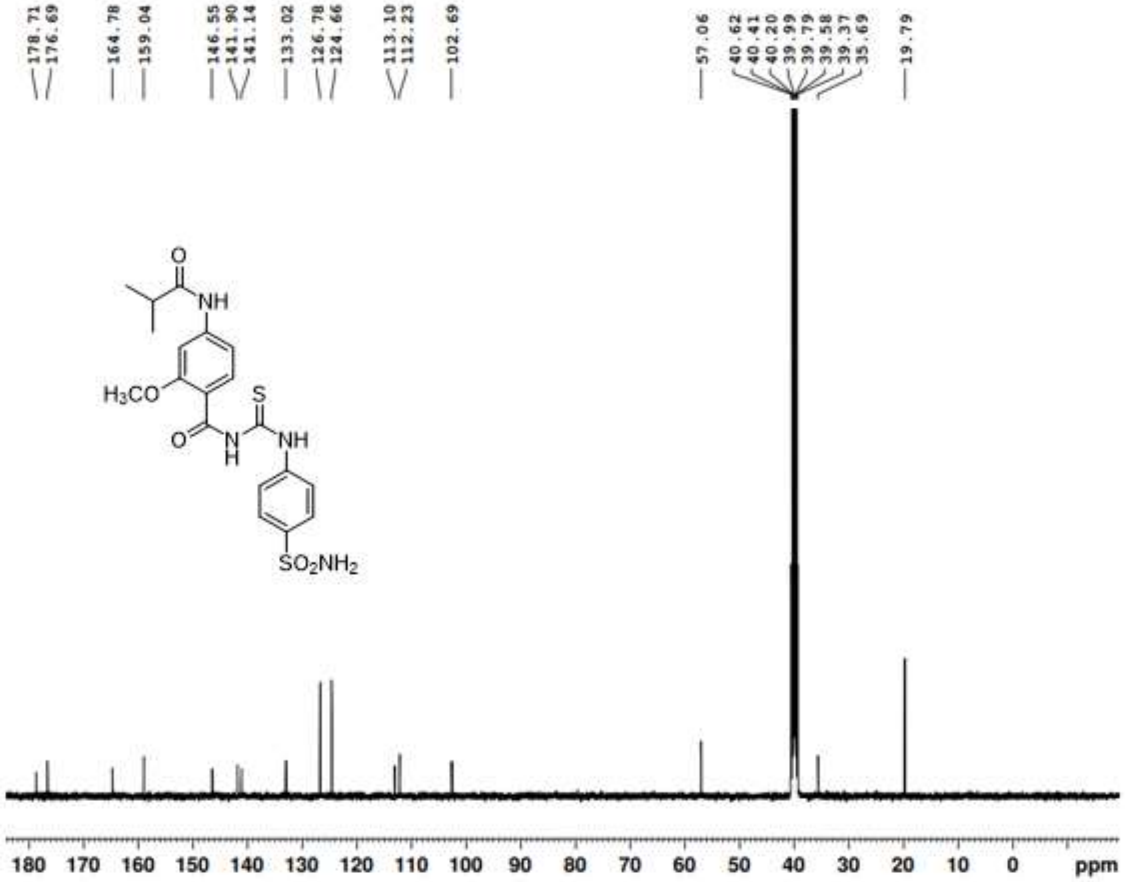

Figure 10: ^{13}C-NMR spectrum of 4-Isobutyramido-2-methoxy-N-[(4-sulfamoyl phenyl)carbamothioyl] benzamide **(12b)**

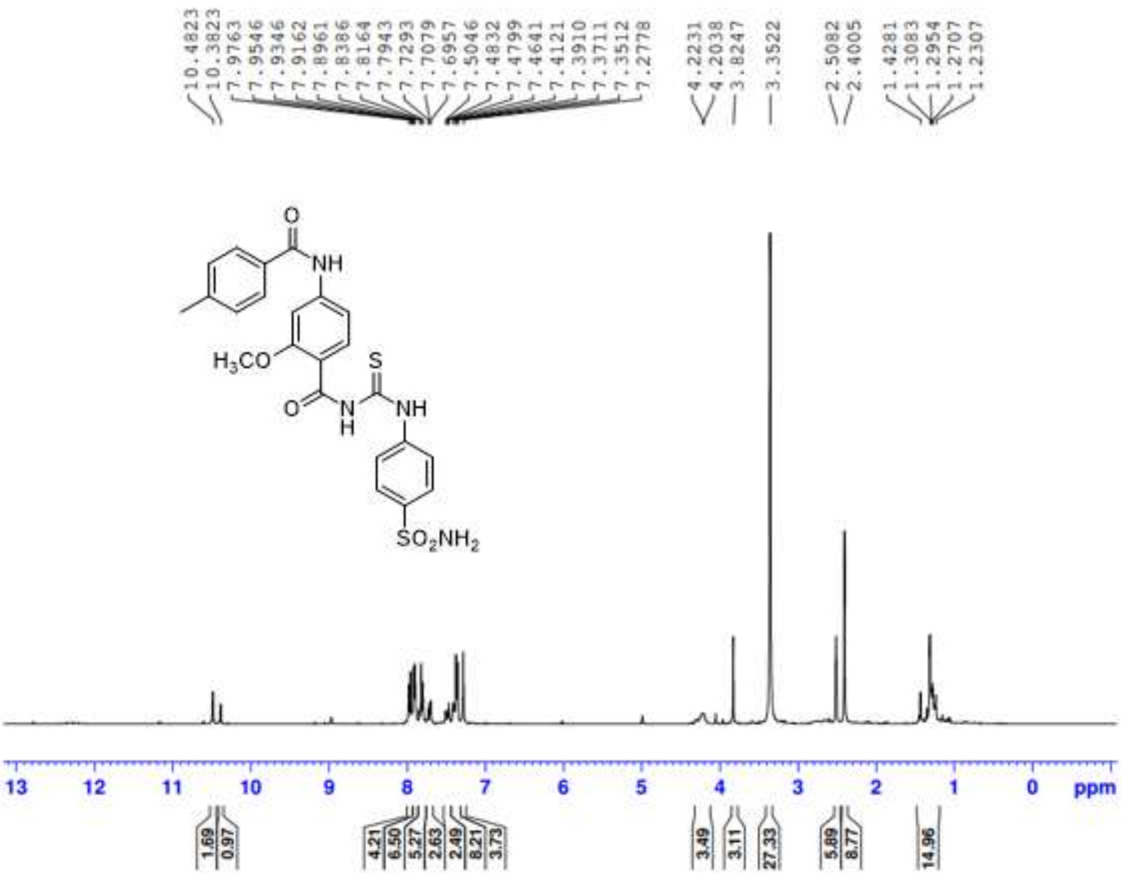

Figure 11: ^{1}H-NMR spectrum of 2-Methoxy-4-(4-methylbenzamido)-N-[(4-sulfamoyl phenyl)carbamothioyl] benzamide **(12c)**

Information Classification: General

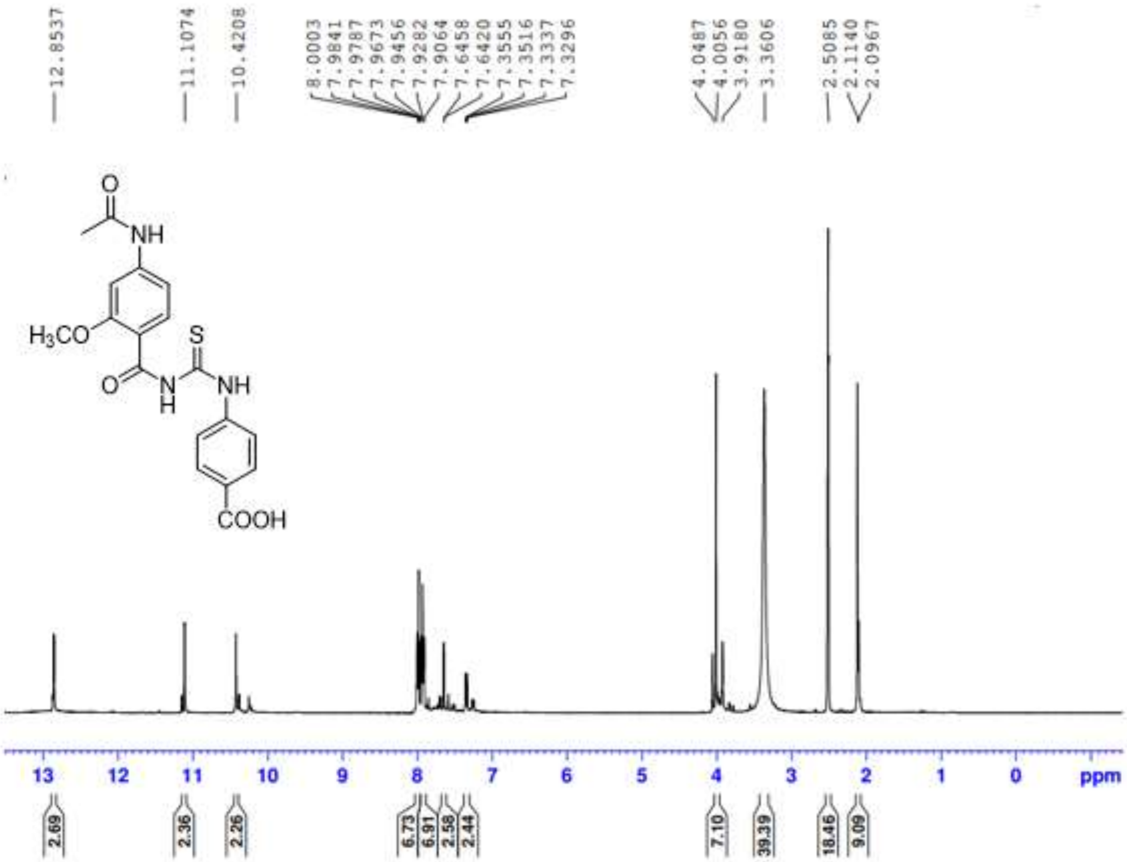

Figure 12: ^{1}H-NMR spectrum of 4-[3-(4-Acetamido-2-methoxybenzoyl)thioureido]benzoic acid **(13a)**

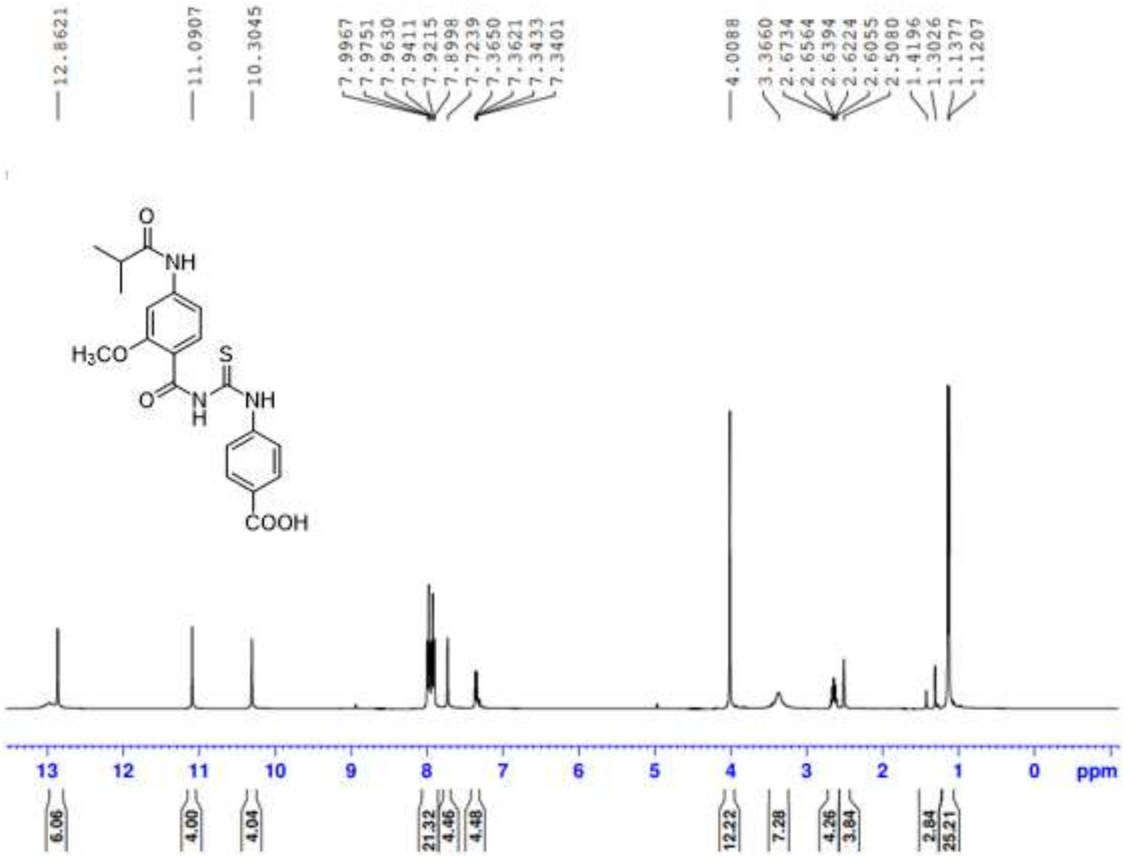

Figure 13: ^{1}H-NMR spectrum of 4-[3-(4-Isobutyramido-2-methoxybenzoyl)thioureid])benzoic acid **(13b)**

Information Classification: General

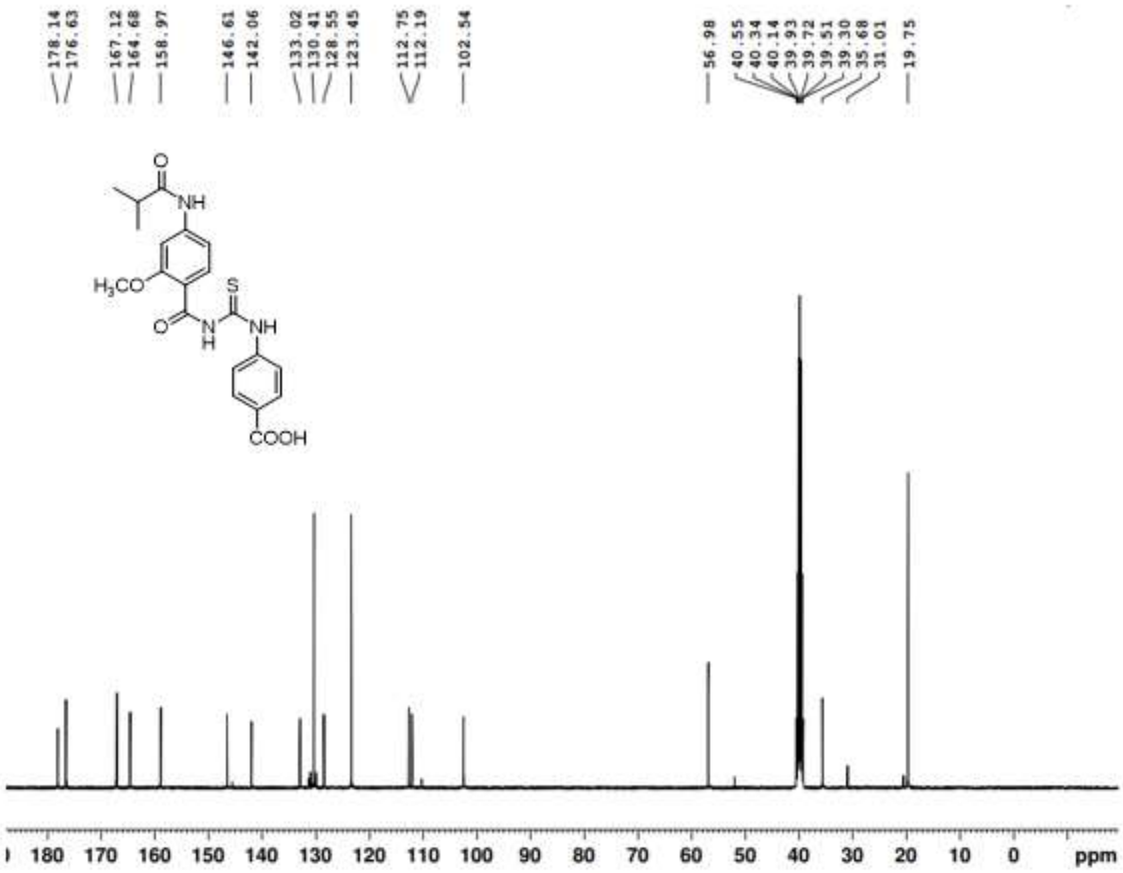

Figure 14: ^{13}C-NMR spectrum of 4-[3-(4-Isobutyramido-2-methoxybenzoyl)thioureid])benzoic acid **(13b)**

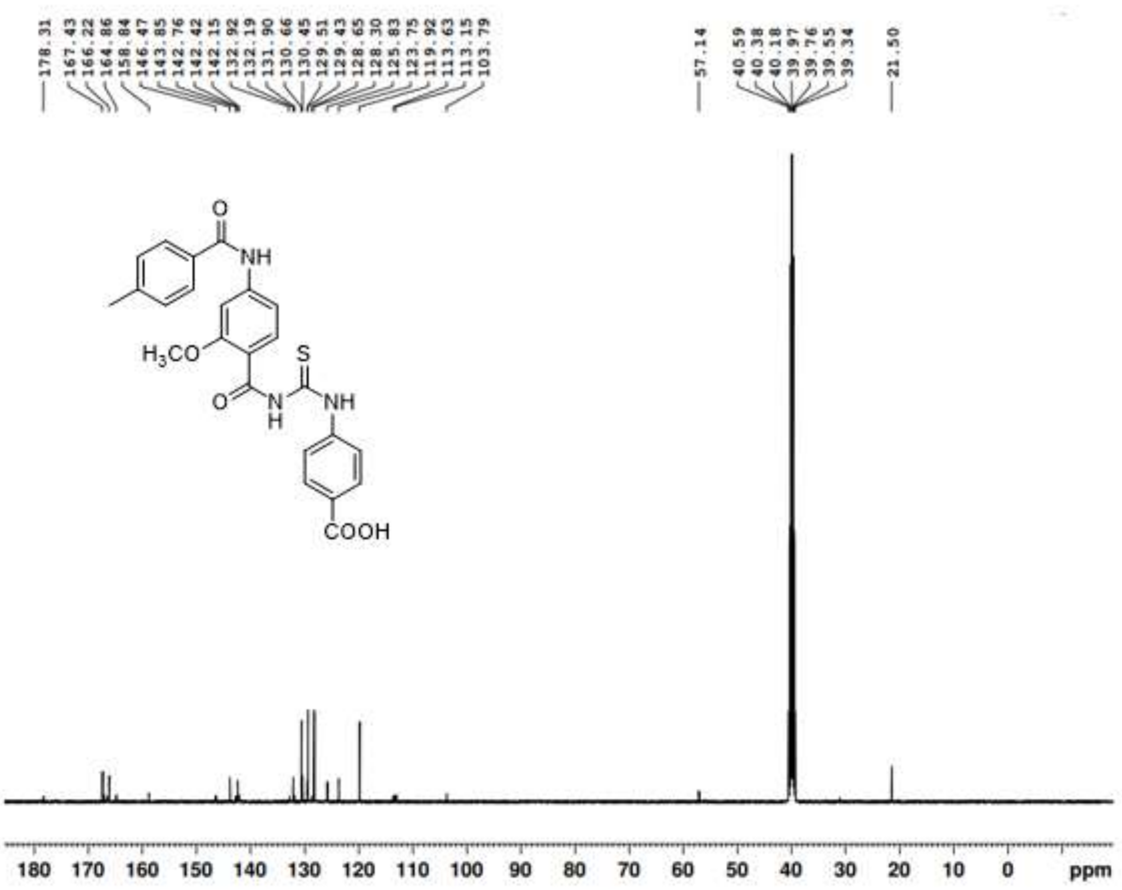

Figure 15: ^{13}C-NMR spectrum of 4-(3-(2-Methoxy-4-(4-methylbenzamido)benzoyl) thioureido]benzoic acid **(13c)**

Information Classification: General

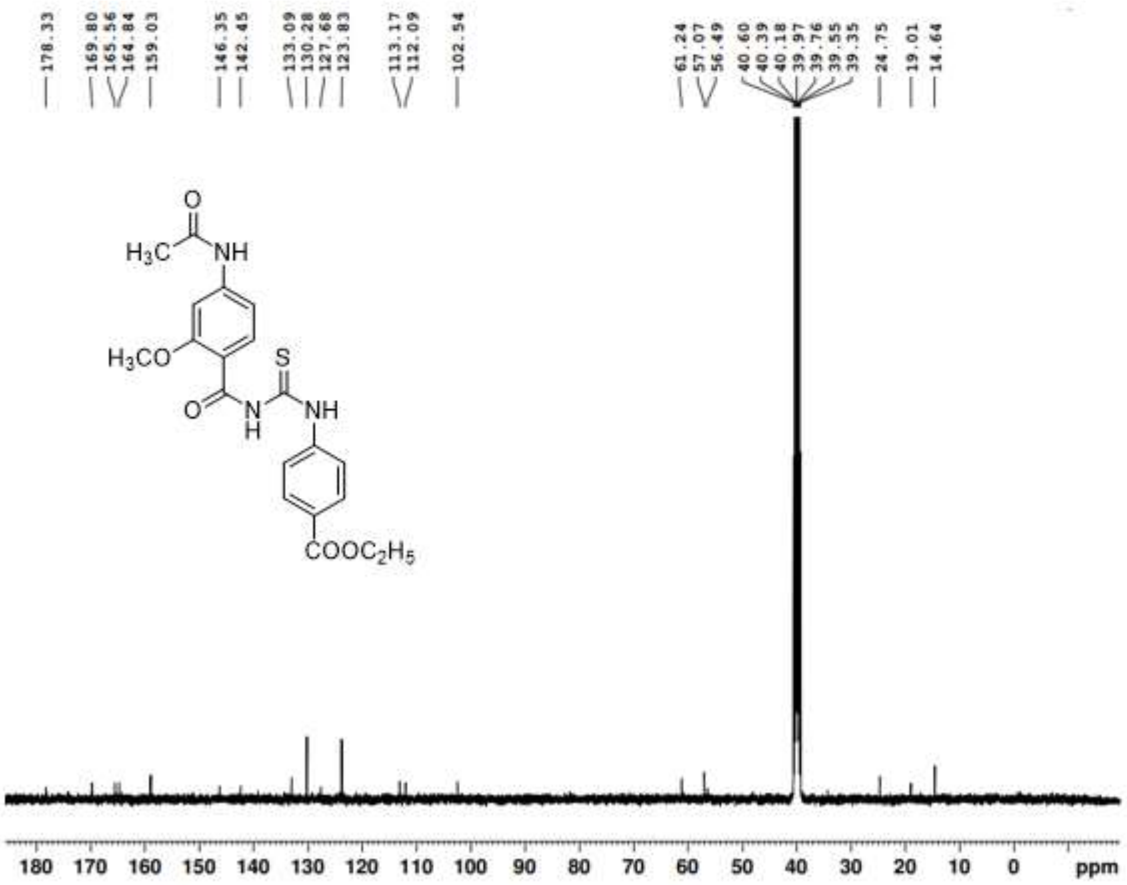

Figure 16: ^{13}C-NMR spectrum of ethyl 4-[3-(4-acetamido-2-methoxybenzoyl)thioureido]benzoate **(14a)**

Information Classification: General

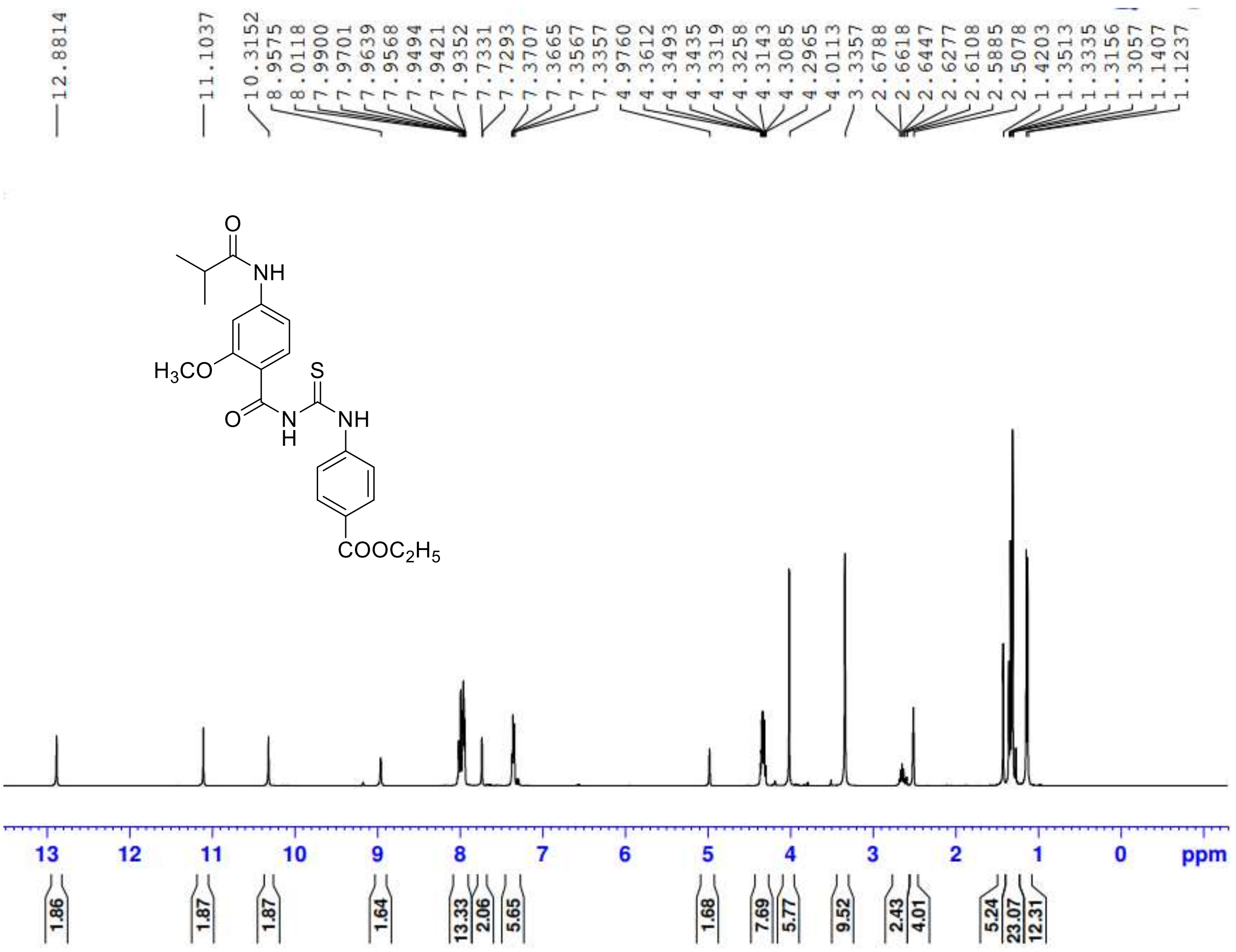

Figure 17: ^{1}H-NMR spectrum of Ethyl 4-[3-(4-isobutyramido-2-methoxy benzoyl)thioureido]benzoate **(14b)**

Information Classification: General

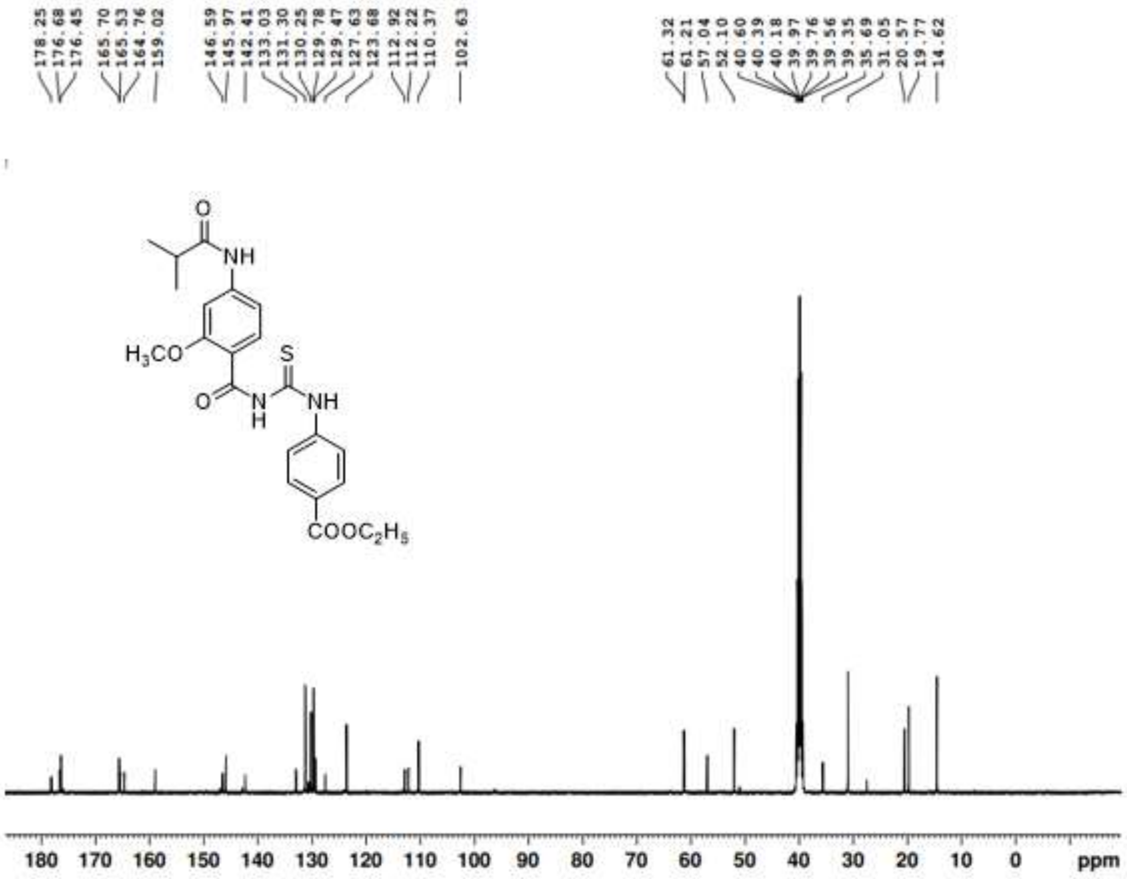

Figure 18: ^{13}C-NMR spectrum of ethyl 4-[3-(4-isobutyramido-2-methoxy benzoyl)thioureido]benzoate **(14b)**

Information Classification: General
